# HUNK inhibits epithelial-mesenchymal transition of CRC via direct phosphorylation of GEF-H1 and activating RhoA/LIMK-1/CFL-1

**DOI:** 10.1038/s41419-023-05849-2

**Published:** 2023-05-16

**Authors:** Xiaoqi Han, Siyuan Jiang, Yinmin Gu, Lihua Ding, Enhao Zhao, Dongxing Cao, Xiaodong Wang, Ya Wen, Yongbo Pan, Xin Yan, Liqiang Duan, Minxuan Sun, Tao Zhou, Yajuan Liu, Hongbo Hu, Qinong Ye, Shan Gao

**Affiliations:** 1 Medical School of Guizhou University, Guiyang, 550025 China; 2 Shanxi Academy of Advanced Research and Innovation, Taiyuan, 030032 China; 3 Zhongda Hospital, School of Life Sciences and Technology, Advanced Institute for Life and Health, Southeast University, Nanjing, 210096 China; 4 Zhongda Hospital, Medical School, Advanced Institute for Life and Health, Southeast University, Nanjing, 210096 China; 5 Department of Medical Molecular Biology, Beijing Institute of Biotechnology, Collaborative Innovation Center for Cancer Medicine, Beijing, 100850 China; 6 Renji Hospital, School of Medicine, Shanghai Jiaotong University, Shanghai, 201200 China; 7 Suzhou Institute of Biomedical Engineering and Technology, Chinese Academy of Sciences, Suzhou, 215163 China; 8 Center for Immunology and Hematology, State Key Laboratory of Biotherapy, National Clinical Research Center for Geriatrics, West China Hospital, Sichuan University, and Collaborative Innovation Center for Biotherapy, Sichuan, 610044 China

**Keywords:** Phosphorylation, Metastasis

## Abstract

Epithelial-mesenchymal transition (EMT) is associated with the invasive and metastatic phenotypes in colorectal cancer (CRC). However, the mechanisms underlying EMT in CRC are not completely understood. In this study, we find that HUNK inhibits EMT and metastasis of CRC cells via its substrate GEF-H1 in a kinase-dependent manner. Mechanistically, HUNK directly phosphorylates GEF-H1 at serine 645 (S645) site, which activates RhoA and consequently leads to a cascade of phosphorylation of LIMK-1/CFL-1, thereby stabilizing F-actin and inhibiting EMT. Clinically, the levels of both HUNK expression and phosphorylation S645 of GEH-H1 are not only downregulated in CRC tissues with metastasis compared with that without metastasis, but also positively correlated among these tissues. Our findings highlight the importance of HUNK kinase direct phosphorylation of GEF-H1 in regulation of EMT and metastasis of CRC.

## Introduction

Colorectal cancer (CRC) is one of the leading causes of cancer-related death worldwide, and more than half of CRC patients develop metastasis [1]. There is no effective therapy for CRC metastatic patients [2]. Cancer cell metastasis requires a complicated cellular process known as epithelial-mesenchymal transition (EMT), during which epithelial cells acquire mesenchymal phenotype, thus resulting in the loss of cell-cell adhesion and polarity, as well as the reorganization of cytoskeleton and reprogramming of gene expression [3, 4]. Numerous studies have shown the critical function of EMT in CRC [5]. Thus, understanding the regulation of CRC cell EMT has the potential to yield new therapeutic opportunities for intervention in CRC [6].

Hormonally Upregulated Neu-associated Kinase (HUNK), a serine-threonine protein kinase regulates cancer cell survival, proliferation and metastasis [7]. However, the controversial conclusions show that HUNK promotes or suppresses metastasis via various signaling pathways by either kinase-independent or -dependent way in breast cancer [8–10]. In other cancer cells, whether HUNK is involved in metastasis and the potential mechanism are unclear. In this study, we found that HUNK suppresses EMT by direct phosphorylation of guanine nucleotide exchange factor H1 (GEF-H1) at serine 645 (S645), a known Rho GTPase activating protein [11, 12], consequently activating RhoA, and a series of phosphorylation of LIM domain kinases (LIMKs)/cofilin 1 (CFL-1) pathway to stabilize F-actin [13]. Collectively, these findings build a linking between HUNK kinase activity and CRC metastasis.

## Results

### HUNK suppresses EMT in CRC cells

**Fig. 1:**
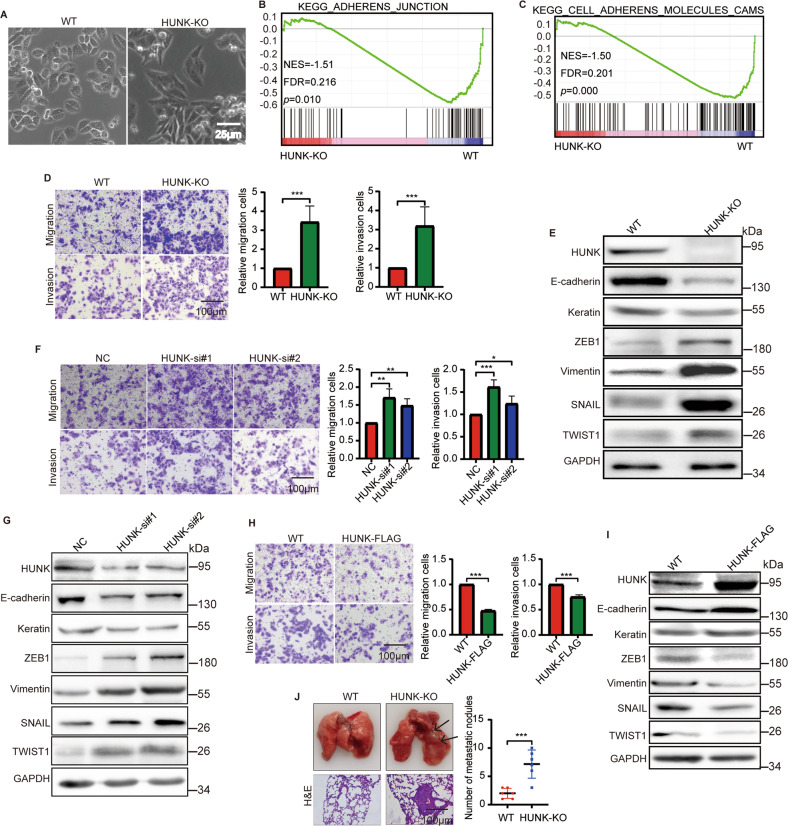
HUNK suppresses EMT of CRC cells. **A** Representative images of the morphology of SW480 WT and HUNK KO cells. Scale bar, 25 μm. GSEA analysis showing the enrichment of “adherens junction” (**B**) and “cell adherens molecules cams” (**C**) between SW480 HUNK KO and WT cells. **D**–**I** Representative micrographs (left) and quantification (right) of the SW480 HUNK KO (**D**), KD (**F**) and OE (**H**) cells in Matrigel-coated or noncoated Transwell assays (*n* = 3); Immunoblot analysis of the indicated proteins in SW480 HUNK KO (**E**), KD (**G**) and OE (**I**) cells. Scale bar, 100 μm. **J** SW480 WT or HUNK KO cells were injected into the tail vein of BALB/c nude mice to establish a lung metastasis model. Representative images (left) of bright-field (top) and H&E staining samples (bottom) from the tumor foci and number (right) of metastatic lesions in mice (*n* = 6), as determined by H&E staining. Scale bars, 100 μm. Results are presented as the mean ± SEM. Two-tailed Student’s *t*-test was used for analyzing the data in (**D**), (**H**) and (**J**). One-way ANOVA with Dunnett’s multiple comparisons test was applied for analyzing the data in (**F**). *p < 0.05, **p < 0.01, and ***p < 0.001.

To explore the potential role of HUNK in metastasis of CRC cells, we generated HUNK knockout (KO) in SW480 cells using CRISPR-Cas9 genomic editing technique (Supplementary Fig. S1A). During the culture of HUNK KO SW480 cells, the mesenchymal-like morphologic features were observed (Fig. 1A), suggesting HUNK negatively regulates EMT. To explore the potential signaling pathways regulated by HUNK, we analyzed RNA expression profiles of HUNK KO cells (Supplementary Fig. S1B, C). Gene Set Enrichment Analysis (GSEA) revealed that HUNK is associated with many signaling pathways, including “adherens junction” and “cell adherens molecules cams” (Fig. 1B, C, Supplementary Fig. S1D), further supporting the potential effects of HUNK on EMT. To functionally validate these observations, we knocked down HUNK by two independent short-interfering RNAs (siRNAs), and overexpressed HUNK using lentivirus vector in two CRC cell lines. Then we performed the transwell invasion and migration assays. Both HUNK KO and knockdown (KD) led to a significant increase in migration and invasion of both SW480 and RKO cells (Fig. 1D, F, Supplementary Fig. S1E). In contrast, the overexpression (OE) of HUNK decreased the invasion and migration ability of these two cell lines (Fig. 1H and Supplementary Fig. S1G). Correspondingly, the expression levels of EMT markers, including E-cadherin, Keratin, ZEB1, vimentin, SNAIL and TWIST1 were detected by immunoblot (IB). The results revealed that the decreased E-cadherin as well as Keratin and increased ZEB1, vimentin, SNAIL as well as TWIST1 were observed in HUNK KO or KD cells compared with the control cells (Fig. 1E, G, Supplementary Fig. S1F). While HUNK OE reduced ZEB1, vimentin, SNAIL as well as TWIST1 and enhanced E-cadherin as well as Keratin (Fig. 1I and Supplementary Fig. S1H). Moreover, characterization of HUNK KO cells by immunostaining showed that E-cadherin was more attenuated, and vimentin was more prominent compared with control (Supplementary Fig. S1I), consistent with EMT phenotype. Moreover, we investigated the role of HUNK in CRC metastasis in vivo using BALB/C nude mice, Haematoxylin-eosin (H&E) staining showed the number of metastatic nodules in the lungs were significantly increased in the HUNK KO group compared to the control group (Fig. 1J). Taken together, these data indicate that loss of HUNK induces the EMT in CRC and promotes metastasis.

### HUNK interacts with GEF-H1

**Fig. 2:**
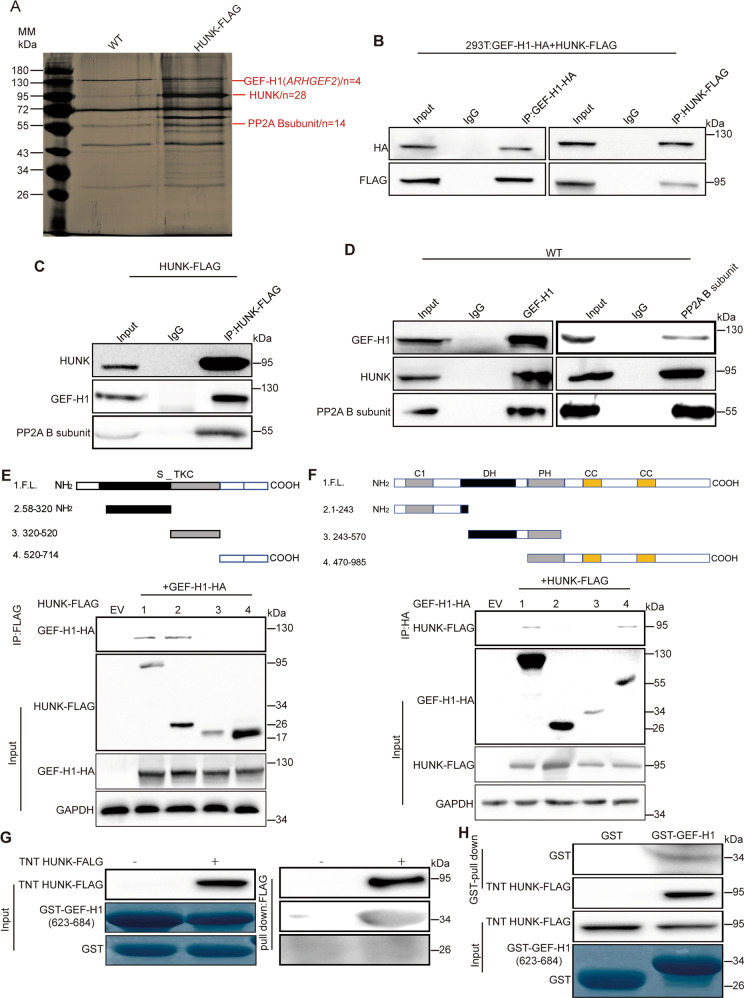
Identification of GEF-H1 as a HUNK binding partner. **A** Visualization of silver-stained protein bands by IP of anti-FLAG beads using total protein extracts from SW480 cells expressing HUNK-FLAG or vector (WT). **B** Lysates of HEK293T cells overexpressing HUNK-FLAG and/or GEF-H1-HA subjected to reciprocal co-IP to detect protein interaction. **C**, **D** Lysates of HUNK OE SW480 cells subjected to co-IP to detect endogenous GEF-H1, PP2A and HUNK-FLAG interaction. Schematic presentation of various human HUNK truncations (**E**) or various human GEF-H1 truncations (**F**) used in binding assays (top); Immunoblot analysis of co-IP from lysates of HEK293T cells overexpressing GEF-H1-HA and/or various HUNK-FLAG truncations (**E**) or HUNK-FLAG and/or various GEF-H1-HA truncations (**F**) (bottom). FL full length, EV empty vector. GAPDH as a loading control. **G**, **H** Pull-down assay for the interaction between cell free translated HUNK and bacterially expressed GST-tagged GEF-H1 (amino acids 623–684).

Given that kinases exert their function mainly by binding to specific substrate proteins [14], we employed affinity purification and mass spectrometry (MS) to identify the proteins that are associated with HUNK. FLAG-tagged HUNK (HUNK-FLAG) was stably expressed in SW480 cells. Cellular extracts were prepared and subjected to affinity purification using an anti-FLAG affinity beads, which were further subjected to liquid chromatography-tandem MS (LC-MS) analysis and led to the identification of protein phosphatase 2-A (PP2A) B subunit and GEF-H1 as the HUNK binding partners (Fig. 2A and Supplementary Fig. S2A and Table S1). The association of PP2A with HUNK or GEF-H1 has been well established [8, 15], consistent with our results. It has been shown that GEF-H1 activates RhoA to promote or inhibit cancer metastasis in a cellular-context-dependent manner [16–18]. We hypothesized that HUNK suppresses EMT via GEF-H1 and focused on GEF-H1. To further validate the physical interaction between HUNK and GEF-H1, we enforced the expression of HUNK-FLAG and GEF-H1-HA in HEK293T cells for reciprocal immunoprecipitation (IP) and confirmed their associations (Fig. 2B). Moreover, co-IP using SW480 cell lysates validated the endogenous association among HUNK, GEF-H1 and PP2A (Fig. 2C, D). We further determine the precision interaction between HUNK and GEF-H1, FLAG-tagged truncated mutants of HUNK were generated and transfected into HEK293T cells. co-IP analysis demonstrated that the 58–320 amino acids of HUNK were required for the interaction of HUNK with GEF-H1 (Fig. 2E). Similarly, the truncated mapping of GEF-H1 showed that a sequence spanning amino acid 470–985 fragment was required for the association with HUNK (Fig. 2F). Furthermore, the pull-down assay results between the bacterially expressed glutathione S-transferase (GST)-GEF-H1 (amino acid 623–684) and cell-freely expressed HUNK showed the direct combination between GEF-H1 and HUNK (Fig. 2G, H). Collectively, these data demonstrate a molecular interface between HUNK and GEF-H1.

### HUNK regulates EMT by RhoA/LIMK-1/CFL-1 signaling

**Fig. 3:**
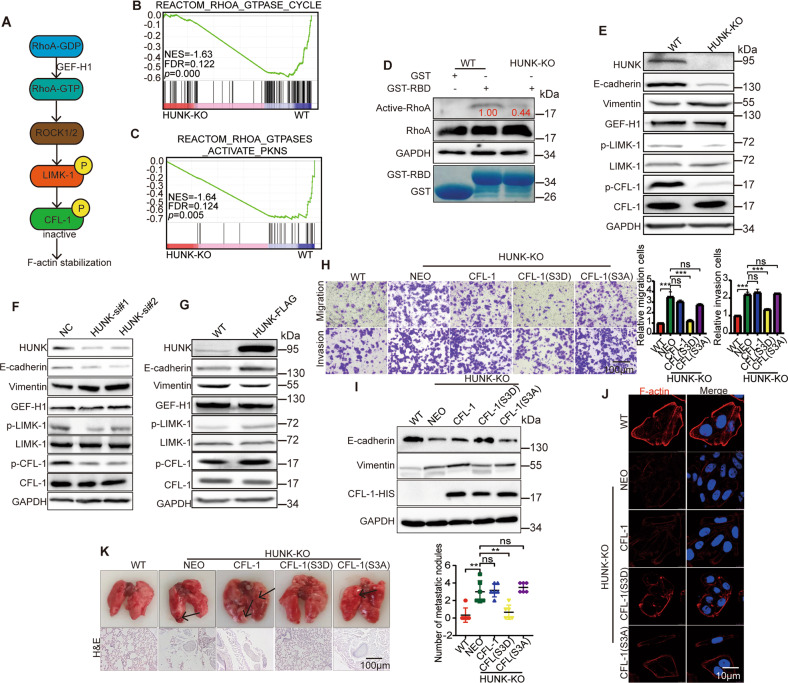
HUNK suppresses EMT of CRC by activating RhoA/LIMK-1/CFL-1 pathway. **A** Schematic presentation of RhoA/LIMK-1/CFL-1 signaling pathway. GSEA analysis showing the enrichment of “RhoA GTPase cycle” (**B**) and “RhoA GTPases activate pkns” (**C**) between SW480 HUNK KO and WT cells. **D** GST pull-down analysis on active RhoA using indicated bacterially expressed GST-RBD domain. The numbers represent the relative intensities of active RhoA normalized to total RhoA and quantified by Image J. Immunoblot analysis of the indicated proteins in SW480 HUNK KO (**E**), KD (**F**) and OE (**G**) cells. **H** Representative micrographs (left) and quantification (right) of the CFL-1(WT), CFL-1 (S3D), CFL-1 (S3A) overexpressed in HUNK KO SW480 cells in Matrigel-coated or noncoated Transwell assays (*n* = 3) Scale bars, 100 μm. **I** Immunoblot analysis of E-cadherin and vimentin in the above-mentioned cells. **J** F-actin was stained using phalloidin-647 in the above-mentioned cells. Nuclei are labeled with 4, 6-diamidino-2-phenylindole (DAPI). The scale bar represents 10 μm. **K** The above-mentioned cells were injected into the tail vein of BALB/c nude mice to establish a lung metastasis model. Number of metastatic lesions in mice (*n* = 6), as determined by H&E staining. Scale bars, 100 μm. Results are presented as the mean ± SEM or SD. One-way ANOVA with Tukey’s multiple comparisons test was applied for analyzing the data in (**H**) and (**K**). **p < 0.01, and ***p < 0.001. ns not significant.

As known that GEF-H1 promotes RhoA activity by accelerating the conversion of GDP-bound RhoA to GTP-bound form [11]. Consequently, GTP-bound RhoA can activate Rho-associated kinase (ROCK), which phosphorylates LIMK-1. CFL-1, a potent actin depolymerizing factor, is one of LIMK’s major substrates. Finally, phosphorylation of CFL-1 (p-CFL-1) by LIMK-1 results in the stabilization of actin filaments and suppresses EMT [18–21]. Thus, we hypothesized HUNK regulates this signaling pathway via GEF-H1 to suppress EMT (Fig. 3A). To explore whether GEF-H1 recapitulates the effects on cell invasion and migration and regulation of the corresponding signaling pathway, we used two independent siRNAs to knock down GEF-H1, which led to a significant increase in the migration and invasion (Supplementary Fig. S2B). IB revealed decreased E-cadherin, p-LIMK as well as p-CFL-1 and increased vimentin expression in GEF-H1 KD cells (Supplementary Fig. S2C). Immunostaining revealed that F-actin depolymerized after GEF-H1 KD (Supplementary Fig. S2D). Overall, these data indicate that GEF-H1 inhibits EMT and regulates this signaling pathway.

We reanalyzed the expression profiles of HUNK KO SW480 cells based on REACTOME database. GSEA identified some metastasis-associated signaling pathways (Supplementary Fig. S3A). Interestingly, gene sets of RhoA active signature were significantly enriched with HUNK, supporting HUNK positively regulates RhoA activity (Fig. 3B, C). We used GST-fused Rho binding domain (RBD), which could specifically bind to active RhoA [22], and immunoprecipitated protein lysate in wild-type and HUNK KO SW480 cells. HUNK KO cells had exhibited a remarkably low level of active RhoA (Fig. 3D). Furthermore, both KO and KD of HUNK cells reduced phosphorylation levels of LIMK-1 and CFL-1, while the total protein levels of these proteins were unaffected (Fig. 3E, F, Supplementary Fig. S3B). In contrast, HUNK OE cells had the opposite effects on this signaling pathway (Fig. 3G, Supplementary Fig. S3C). These data demonstrate that HUNK regulates RhoA/LIMK-1/CFL-1 signaling.

To determine whether RhoA/LIMK/CFL-1 signaling is a key downstream axis of the HUNK/GEF-H1 suppressing the EMT of CRC cells, we next performed rescue experiments. CCG-1423 is a highly selective RhoA inhibitor [23], thus, we explored the blocked effects of CCG-1423 on EMT in HUNK KO and control SW480 cells. Strikingly, CCG-1423 inhibitor promoted invasion as well as migration, and inhibited the phosphorylation of LIMK-1 and CFL-1 in HUNK control cells, but had no effects in HUNK KO cells (Supplementary Fig. S3D, E), suggesting that HUNK negatively regulates EMT via RhoA in CRC cells. Next, we generated CFL-1 wild-type (WT) construct, non-phosphorylatable alanine mutant (S3A) and phosphomimetic aspartic acid mutant (S3D), which were overexpressed in HUNK KO SW480 cells, respectively. As expected, the effects of HUNK KO on transwell assays and the expression of EMT protein makers as well as F-actin were markedly rescued by CFL-1 S3D, but not CFL-1 WT and CFL-1 S3A (Fig. 3H–J). Furthermore, we also explored the effects of CFL-1 in HUNK-mediated cancer metastasis in vivo. As expected, the promoting effects of HUNK KO on SW480 cell metastasis were rescued by CFL-1 S3D OE (Fig. 3K). Altogether, these data demonstrate that HUNK suppresses EMT through the phosphorylated CFL-1.

### HUNK-mediated GEF-H1 phosphorylation suppresses EMT

**Fig. 4:**
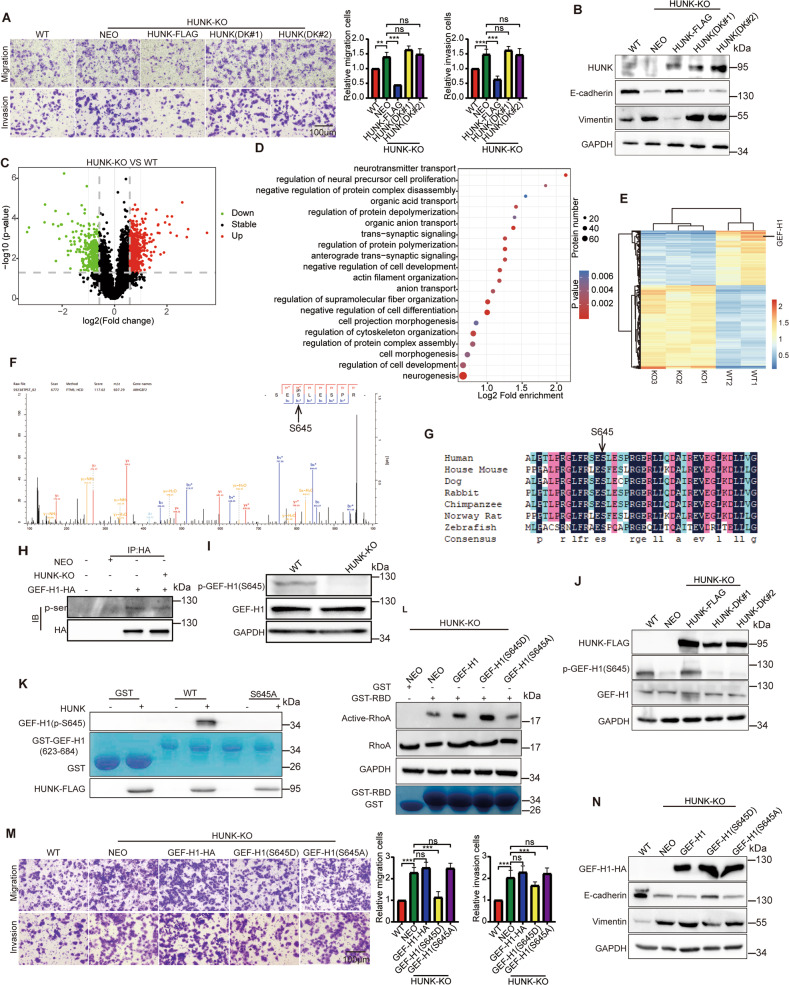
HUNK suppresses EMT in CRC cells through the S645 phosphorylation of GEF-H1. **A** Representative micrographs (left) and quantification (right) of HUNK (WT), HUNK (DK#1), HUNK (DK#2) overexpressed in HUNK KO SW480 cells in Matrigel-coated or noncoated Transwell assays (*n* = 3). Scale bars, 100 μm. **B** Immunoblot analysis of the indicated proteins in the above-mentioned cells. **C** Volcano plot showing the downregulated 274 (green) and upregulated 420 (red) phosphorylation sites of quantitative phosphoproteomic between HUNK KO and WT SW480 cells. **D** Top 20 enriched GO terms (*p* < 0.05) for differential phosphorylation proteins sorted by Log_2_ Fold Enrichment. The color intensity indicates the p values. The size of the circle represents the number of enriched proteins in the term. **E** Heat map shows differential phosphorylation proteins upon HUNK KO in SW480 cells. The upregulated and downregulated genes in HUNK cells were colored in red and blue, respectively. **F** MS analysis of tryptic peptide and ion fragmentation identifying phosphorylation of ph-Ser645. **G** Peptide alignment of GEF-H1 (amino acids 645) in various species. **H** Lysates of HUNK KO and control SW480 cells overexpressing GEF-H1-HA were subjected to co-IP, and immunoblot using pan-phosphorylation serine antibody. **I** Immunoblot analysis of p-GEF-H1(S645) antibody in HUNK KO SW480 cells. **J** Immunoblot analysis of p-GEF-H1(S645) antibody in HUNK (WT), HUNK (DK#1), HUNK (DK#2) overexpressed in HUNK KO SW480 cells. **K** In vitro kinase analysis of recombinant GST-GEF-H1(amino acids 623–684) and active human HUNK proteins were incubated with GST-GEF-H1 for kinase reaction. Phosphorylated proteins were separated by SDS-PAGE and analyzed by immunoblot. Loading controls were shown in the bottom panels. **L** GST pull-down analysis on active RhoA using bacterially expressed GST-RBD domains for HUNK KO SW480 cells transfected with the indicated plasmids. **M** Representative micrographs (left) and quantification (right) of above-mentioned cells in Matrigel-coated or noncoated Transwell assays (*n* = 3), Scale bars, 100 μm. **N** Immunoblot analysis of the indicated proteins in above-mentioned cells. Results are presented as the mean ± SEM. One-way ANOVA with Tukey’s multiple comparisons test was applied for analyzing the data in (**A**) and (**M**). **p < 0.01, and ***p < 0.001. ns not significant.

HUNK is a serine/threonine kinase, and plays an important role in many cancer types, including CRC [7, 24]. We speculated that HUNK exerts its biological functions through its kinase activity. To test this hypothesis, we generated two dead kinase mutants (DKs), whose 91 lysine of kinase catalytic domain was replaced by methionine (K91M) or arginine (K91R) with the loss of kinase activity [9, 25]. We enforced these constructs in HUNK KO cells. The effects of HUNK KO on EMT have been reversed by the expression of HUNK but not two HUNK DKs (Fig. 4A, B), suggesting HUNK regulates EMT dependent on its kinase activity. To identify the potential substrates that were phosphorylated by HUNK, we performed the quantitative phosphoproteomic analysis with a tandem mass tag labeling approach in SW480 KO and control cells, which revealed phosphorylation changes of hundreds of proteins (Fig. 4C and Supplementary Dataset 1). Furthermore, gene ontology (GO) analysis revealed that these differential phosphorylation proteins were mainly enriched in signaling pathways such as “regulation of actin filament organization” and “regulation of cytoskeleton organization” (Fig. 4D), supporting that HUNK kinase activity is involved in EMT. Notably, GEF-H1 was a top phosphorylation candidate and S645 was a potentially phosphorylated site (Fig. 4E, F), which was largely conserved among vertebrates (Fig. 4G), indicating an evolutionarily conserved role in the regulation of the GEF-H1 by HUNK. We first examined the serine phosphorylation level of GEF-H1 using anti-pan-phosphorylation serine antibody, which showed that serine phosphorylation levels of GEF-H1 in HUNK KO cells were low compared to that in control cells (Fig. 4H). We next sought to explore whether HUNK directly phosphorylated GEF-H1. We generated a polyclonal rabbit antibody against the phosphorylated S645 site of GEF-H1, which detected a specific signal in HUNK control cells, but not in HUNK KO cells (Fig. 4I), suggesting that this phosphorylation was specific and GEF-H1 was phosphorylated by HUNK at S645. Furthermore, the phosphorylated S645 level of GEF-H1 was restored by HUNK OE but not HUNK KDs (Fig. 4J). Finally, we performed in vitro kinase assays and found that HUNK phosphorylated (GST)-GEF-H1 but not GST alone. Mutant of S645A of GEF-H1 abolished the phosphorylation signal (Fig. 4K). These data demonstrated that S645 of GEF-H1 is a major site subjected to HUNK phosphorylation. Furthermore, we verified the effects of phosphorylated S645 site of GEF-H1 on RhoA activity, which revealed that S645D mutant, but not S645A and WT constructs rescued RhoA activity level (Fig. 4L). Next, we also explored the effects of phosphorylated S645 site of GEF-H1 on SW480 cell EMT, which showed that S645D mutant, but not S645A and WT constructs rescued EMT of HUNK KO cells (Fig. 4M, N). Collectively, these results corroborate the notion that HUNK directly phosphorylates GEF-H1 at S645 site that negatively regulates EMT.

### Pharmacological inhibition of HUNK mimics the effects of HUNK depletion

**Fig. 5:**
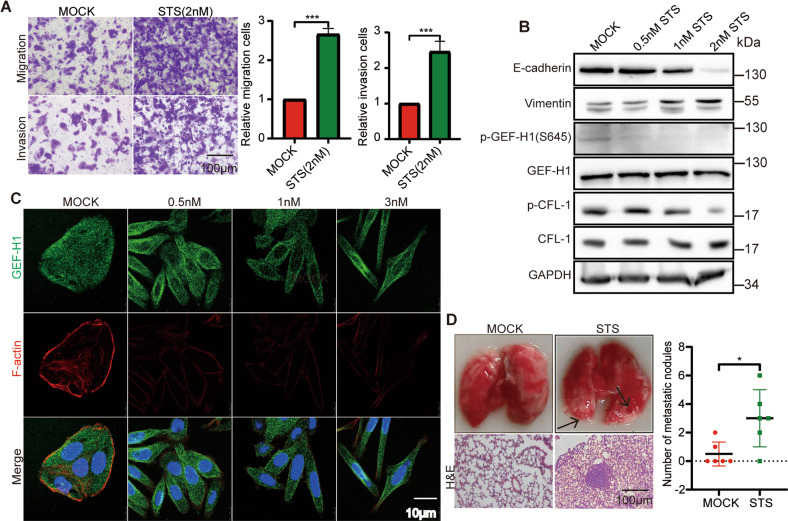
The pharmacological inhibition of HUNK accelerates metastasis of CRC cells. **A** Representative micrographs (left) and quantification (right) of DMSO (MOCK)- and STS-treated SW480 cells in Matrigel-coated or noncoated Transwell assays (*n* = 3). Scale bars, 100 μm. **B** Immunoblot analysis of the indicated proteins in MOCK- and STS-treated SW480 cells. **C** Representative images of F-actin and GEF-H1 stained in STS concentration gradient treatment SW480 cells. Nuclei are labeled with DAPI. Scale bars, 10 μm. **D** MOCK and STS treatment cells (*n* = 6) were injected into the tail vein of BALB/c nude mice to establish a lung metastasis model. Number of metastatic lesions in mice (*n* = 6), as determined by H&E staining. Scale bars, 100 μm. Results are presented as the mean ± SEM or SD. Two-tailed Student’s *t*-test was used for analyzing the data in (**A**) and (**D**). *p < 0.05 and ***p < 0.001.

As shown that HUNK suppresses EMT dependent on its kinase activity, we used HUNK inhibitor to perform proof-of-principle experiments, testing the effects of HUNK inhibition on EMT and the corresponding signaling pathway. Staurosporine (STS) is a potential inhibitor of HUNK [26, 27]. The transwell assays revealed that SW480 cells had the significantly increased invasion and migration in STS treatment cells compared with DMSO-treated cells (Fig. 5A). Furthermore, the expression levels of the p-GEF-H1, p-CFL-1, E-cadherin and vimentin showed a dose-dependent pattern with STS treatment (Fig. 5B), which phenocopied HUNK depletion. Immunostaining revealed that STS-treated cells underwent mesenchymal-like change and F-actin depolymerization with a dose-dependence (Fig. 5C). We further tested the effects of this agent on cancer metastasis in vivo. The intravenous SW480 cells from STS-treated mice markedly formed more lung metastatic nodules compared with the control mice (Fig. 5D). Taken together, these data demonstrate that pharmacological inhibition of HUNK inhibits the phosphorylation of GEF-H1 S645 and consequently promotes metastasis.

### HUNK is clinically associated with the phosphorylated S645 of GEF-H1

**Fig. 6:**
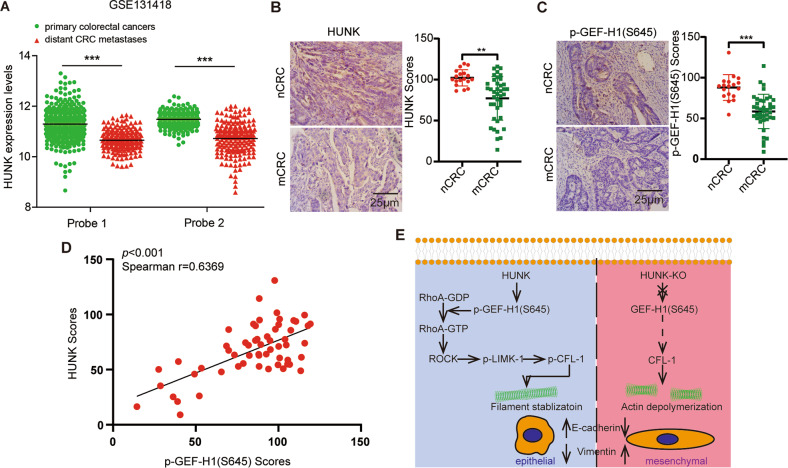
The correlation between HUNK and p-GEF-H1(S645) in the CRC tissues. **A** The expression levels of two probes for HUNK in primary and distant of CRC patients from the indicated GEO dataset. Representative images (left) and quantification (right) of HUNK (**B**) and p-GEF-H1(S645) expression (**C**) in non-metastatic CRC (nCRC) and metastatic CRC (mCRC) primary tissues. Scale bars, 25 μm. **D** Correlation analysis of HUNK and p-GEF-H1(S645) expression. **E** Proposed model for the HUNK/GEF-H1 axis suppressing the EMT of CRC. Two-tailed Student’s *t*-test was used for analyzing the data in (**A**–**C**). Spearman’s correlation was used for analyzing the data in (**D**). **p < 0.01, and ***p < 0.001.

To understand the clinical role of HUNK in CRC, we performed bioinformatics analyses of the transcriptional levels of HUNK for CRC patients from The Cancer Genome Atlas (TCGA) database. The transcriptional *HUNK* levels significantly increased in CRC tissues compared to adjacent tissues (Supplementary Fig. S4A). The transcriptional levels of *HUNK* were similar between the N0 and N1 tissues as well as the M0 and M1 tissues (Supplementary Fig. S4B, C). In addition, a Gene Expression Omnibus (GEO) dataset showed that the transcriptional levels of *HUNK* were lower in metastatic samples than in primary samples (Fig. 6A). We further evaluated the protein expressions of HUNK and p-GEF-H1 S645 in primary CRC tissues without (nCRC) or with metastasis (mCRC) (Supplementary Table. S2). The results revealed that the expression levels of HUNK and p-GEF-H1 S645 were increased in nCRCs compared with mCRCs (Fig. 6B, C). Moreover, a correlation analysis showed that the HUNK protein levels were positively correlated with the GEF-H1 phosphorylation S645 expression levels (Fig. 6D), suggesting that HUNK phosphorylates S645 of GEF-H1 in CRC patients. Overall, the results indicate that HUNK is a potential prognostic marker and therapeutic target for CRC.

## Discussion

HUNK plays an important role in cancer cell proliferation, survival, as well as metastasis, and is an emerging therapeutic target for cancer treatments [7, 8, 10, 28–32]. Clinically, we showed that HUNK protein levels are downregulated in metastatic CRC tissues in contrast to primary CRC tissues. Furthermore, we found HUNK KO decreased, whereas HUNK OE increased the proliferation and colony formation of CRC cells (Supplementary Fig. S5B–F), but the underlying mechanism of HUNK-mediated CRC cell proliferation remains to be explored in future study. Wnt/β-catenin signaling pathway is abnormally activated in CRC [33], we further checked whether HUNK affects the activity of this signaling pathway, and found that HUNK did not affect β-catenin expression level and localization in HUNK KO cells (Supplementary Fig. S6A–C). The previous studies showed that HUNK regulates cancer metastasis via different mechanisms [8, 10]. However, we found that HUNK suppresses metastasis of CRC cells via activating GEF-H1 by direct phosphorylation at S645 site, which promotes GTP-bound RhoA, thus consequently leading to a sequential phosphorylation event of ROCK/LIMK-1/CFL-1 [11, 13]. In consistent with our study, it has been demonstrated that HUNK suppresses cancer metastasis by competitively binding to CFL-1 and protecting dephosphorylation of CFL-1 by PP2A [8]. Indeed, we also identified that PP2A is physically associated with HUNK and GEF-H1. These data suggests that PP2A plays the dual function in promoting cancer metastasis. How PP2A plays such function under either cellular-context dependence or cell type dependence need to be further clarified in detailed. As a kinase, it has been shown that functions of HUNK are dependent on its kinase activity, and a few substrates including EGFR have been identified [9, 10, 27, 34, 35]. Our study clearly provided a new direct phosphorylation substrate, i.e., GEF-H1. The conflicting conclusions for HUNK-mediated cancer metastasis are probably resulted from option of the signaling pathway, cellular-context dependence, or cell-type specificity in either cell proliferation or metastasis. The precise molecular and cellular mechanisms of these discrete observations require further in-depth study.

Small guanosine triphosphatase (GTPase) family plays an important role in cell proliferation, cytoskeletal organization, cell polarity, etc., many of which are deregulated in various cancer types [36]. Unlike higher mutations of Ras family in cancer, very few mutations in Rho family are identified. However, the deregulated expression or activity of Rho family including RhoA appears to mediate key function in cancer [37]. GEF-H1 is a major activating regulator of RhoA and an attractive target in cancer treatment [11]. The primary mechanism of GEF-H1 regulation in cancer cells is via phosphorylation. Numerous studies have shown that a few phosphorylation sites of GEF-H1 mediate the different signaling pathways and/or the binding selections of downstream effectors to function in cancer. For example, phosphorylation of GEF-H1 at threonine 678 by ERK1/2 meditates the activation of RhoA [12, 38], which consequently activates ROCK-myosin in response to tumor necrosis factor stimulation [12]. In contrast, ERK1/2 can also inhibit GEF-H1 by phosphorylating S959 to regulate cancer cell metastasis [39]. Here, we demonstrated that S645 phosphorylation of GEF-H1 by HUNK is an important suppressor for CRC cell EMT. Furthermore, RhoA inactivating regulator, i.e., Rho GTPase activating protein 29 (ARHGAP29), has been shown to regulate cancer cell metastasis via RhoA/LIMK/CFL-1 signal [40]. Clearly, our study provides insight on GEF-H1-mediated EMT and expands the knowledge in regulation of EMT. This will improve our understanding of the mechanistic, functional, and pathological roles of GEF-H1 in cancers and will contribute to therapeutic perspectives for cancer therapy. It is desirable to explore that how these phosphorylation sites coordinate to function in cancer.

Overall, our data reveal that HUNK directly phosphorylates S645 of GEF-H1, consequently activating RhoA/LIMK-1/CFL-1 and stabilizing F-actin to inhibit EMT in CRC (Fig. 6E). This study provides the underlying mechanism for HUNK inhibited EMT and metastasis, thus pave a way for targeting cancer metastasis.

## Supplementary information

Dataset 1

Original Data File

aj-checklist

Supplementary Information -final

## Data Availability

The data and materials during this study are available from the corresponding author on reasonable request.
