# Supplementary material for: HUNK inhibits epithelial-mesenchymal transition of CRC via direct phosphorylation of GEF-H1 and activating RhoA/LIMK-1/CFL-1
Source: Cell Death Dis. 2023 May 16;14(5):327. doi: 10.1038/s41419-023-05849-2 (PMC10188538; doi:10.1038/s41419-023-05849-2)
Supplement: Supplementary file 2 — Original Data File [file 41419_2023_5849_MOESM2_ESM.pdf]

Fig 1E

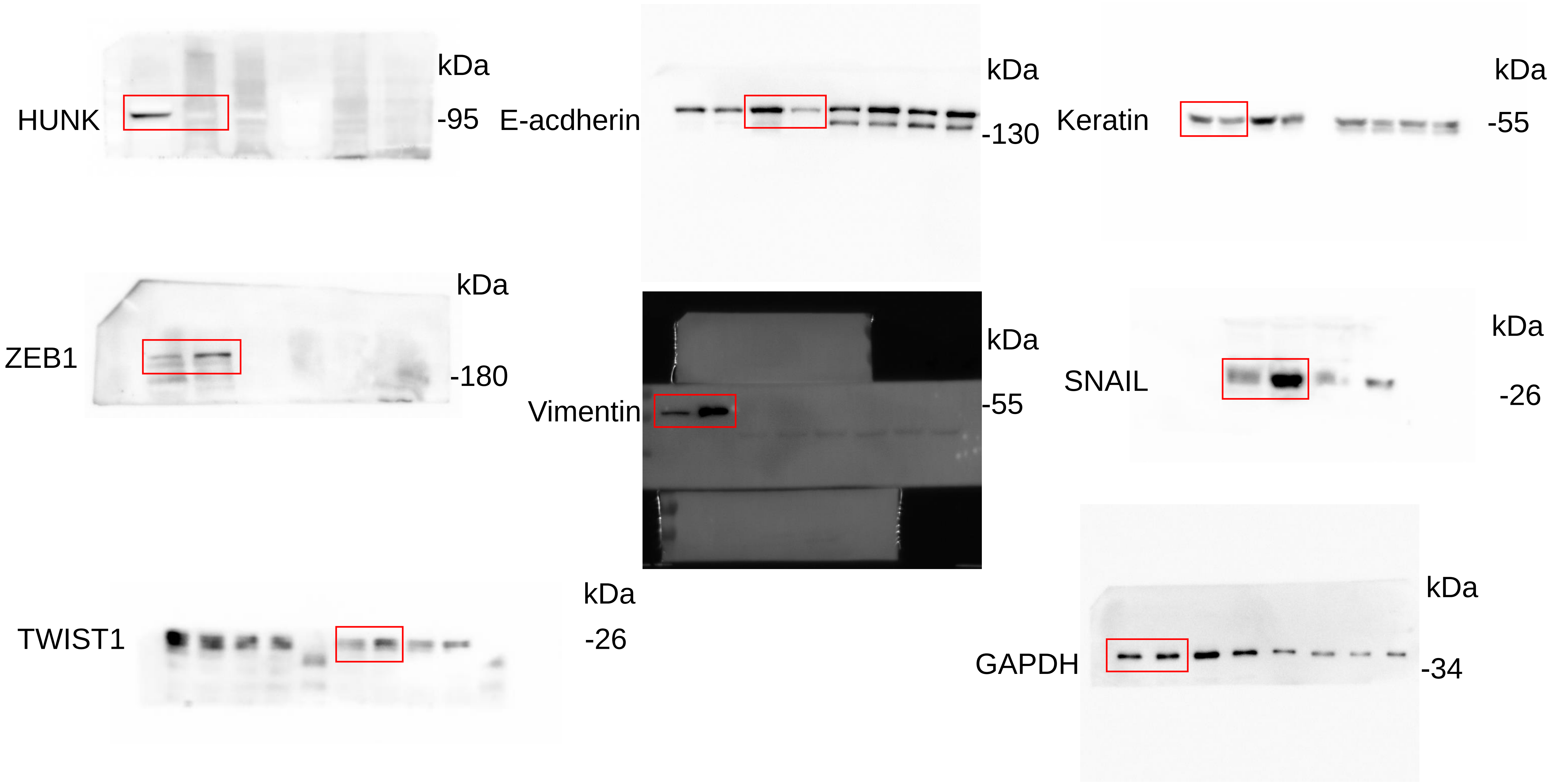
HUNK
kDa
-95
E-acdherin
kDa
-130
Keratin
kDa
-55
ZEB1
kDa
-180
Vimentin
kDa
-55
SNAIL
kDa
-26
TWIST1
kDa
-26
GAPDH
kDa
-34

Fig 1G

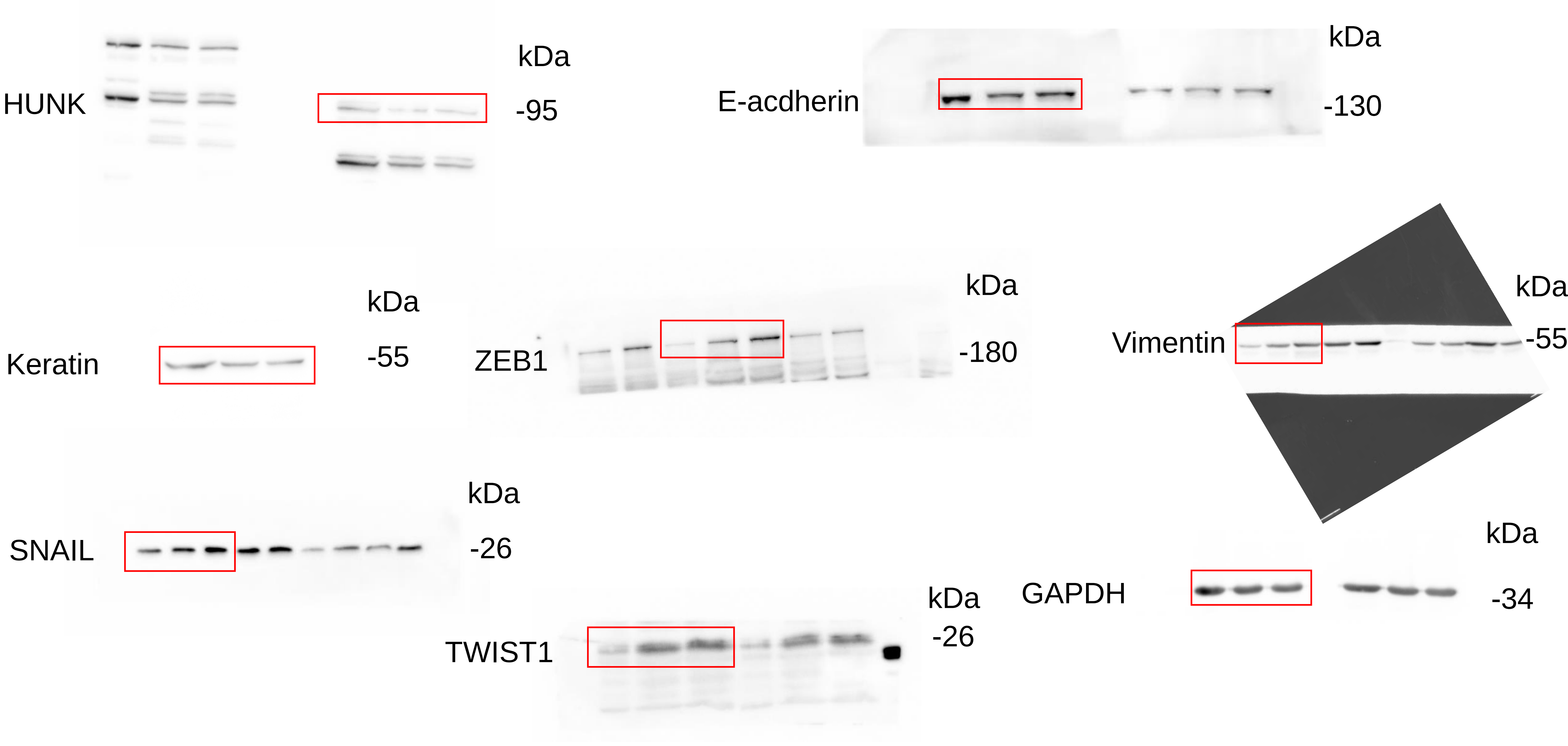
HUNK
kDa
-95
E-acdherin
kDa
-130
Keratin
kDa
-55
ZEB1
kDa
-180
Vimentin
kDa
-55
SNAIL
kDa
-26
TWIST1
kDa
-26
GAPDH
kDa
-34

Fig 1I

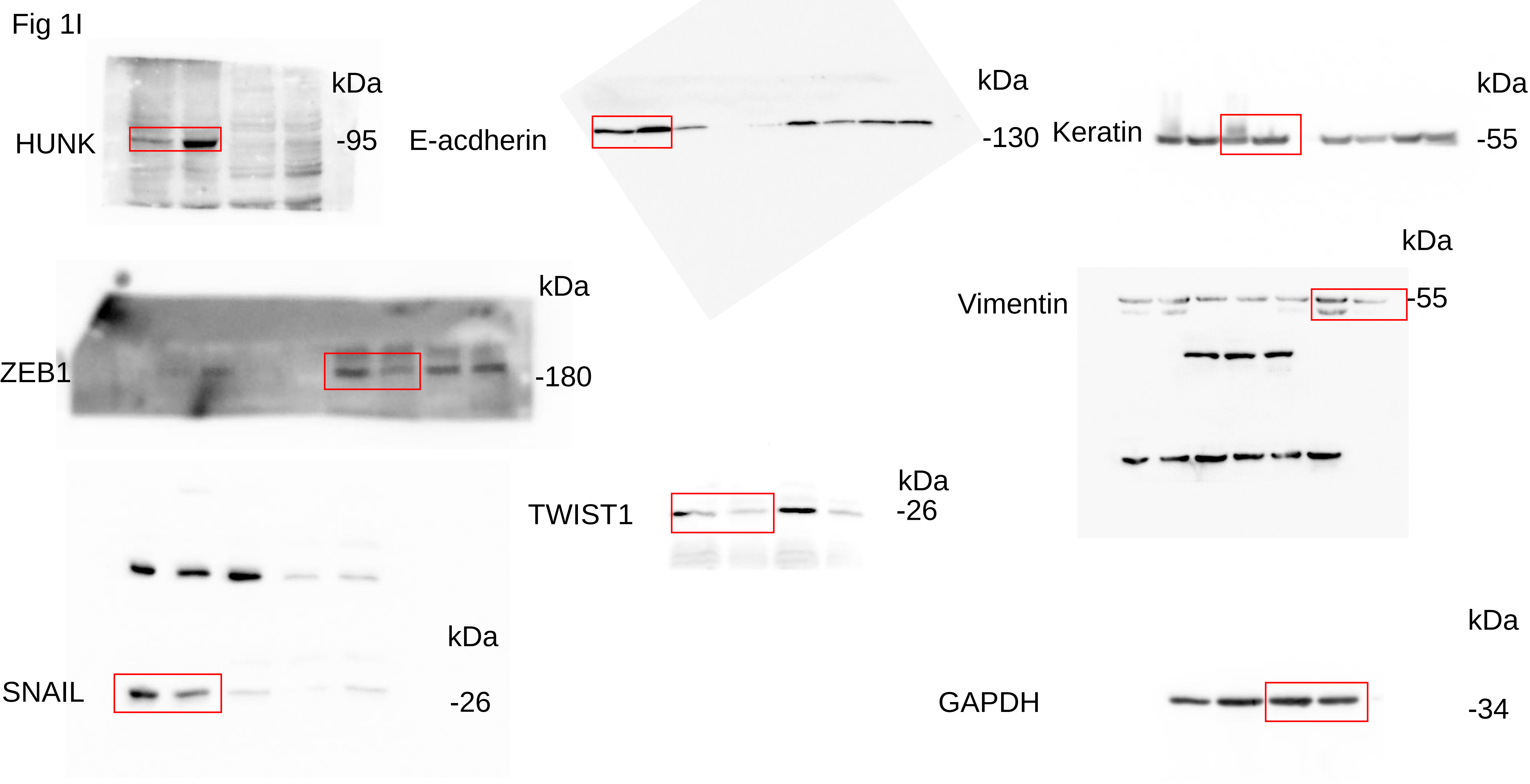
HUNK
kDa
-95
E-acdherin
kDa
-130
Keratin
kDa
-55
ZEB1
kDa
-180
Vimentin
kDa
-55
TWIST1
kDa
-26
SNAIL
kDa
-26
GAPDH
kDa
-34

Fig 2B

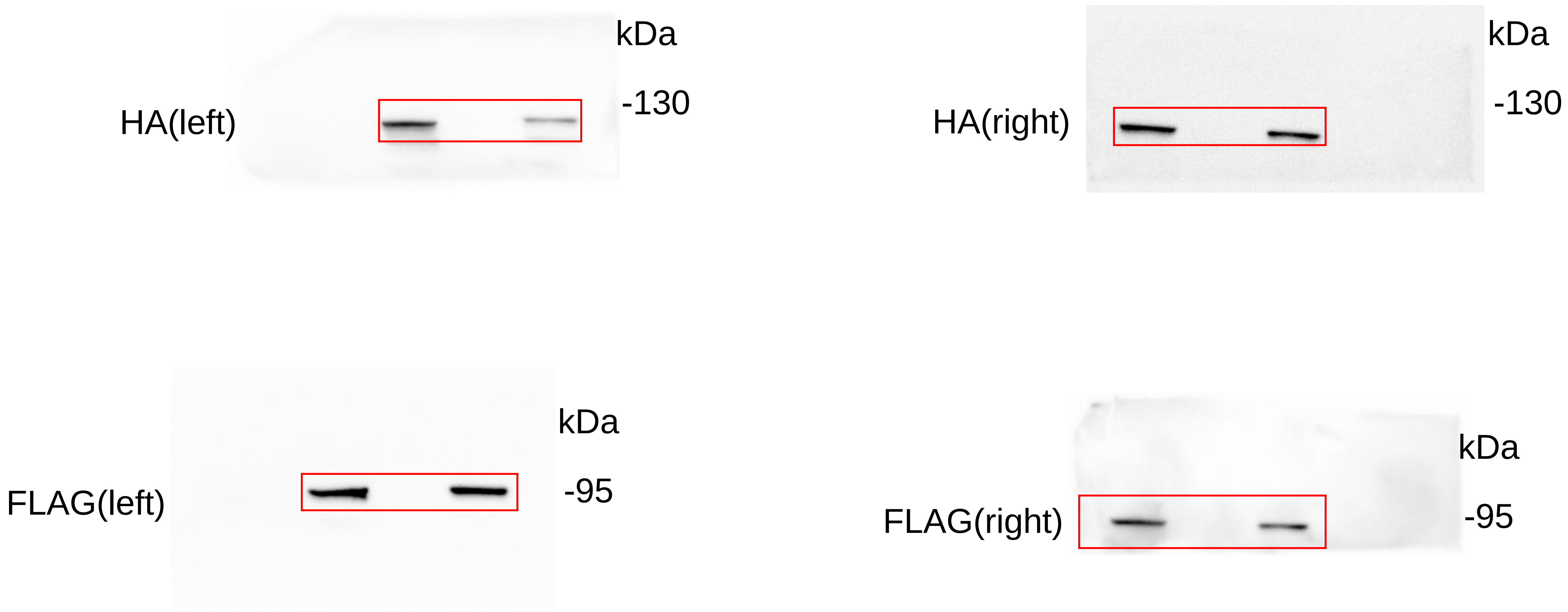

HA(left)
kDa
-130
HA(right)
kDa
-130
FLAG(left)
kDa
-95
FLAG(right)
kDa
-95

Fig 2C

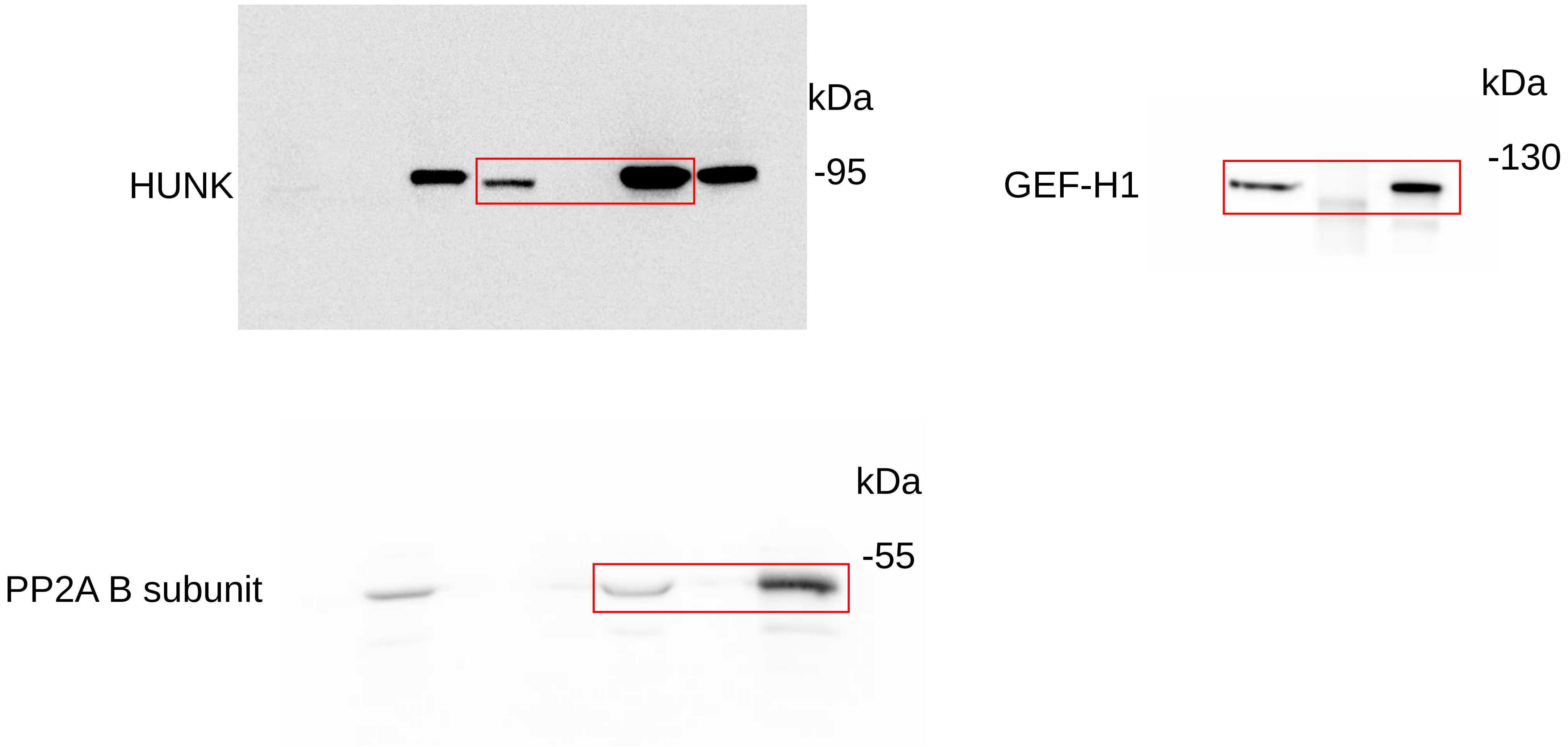

Fig 2D

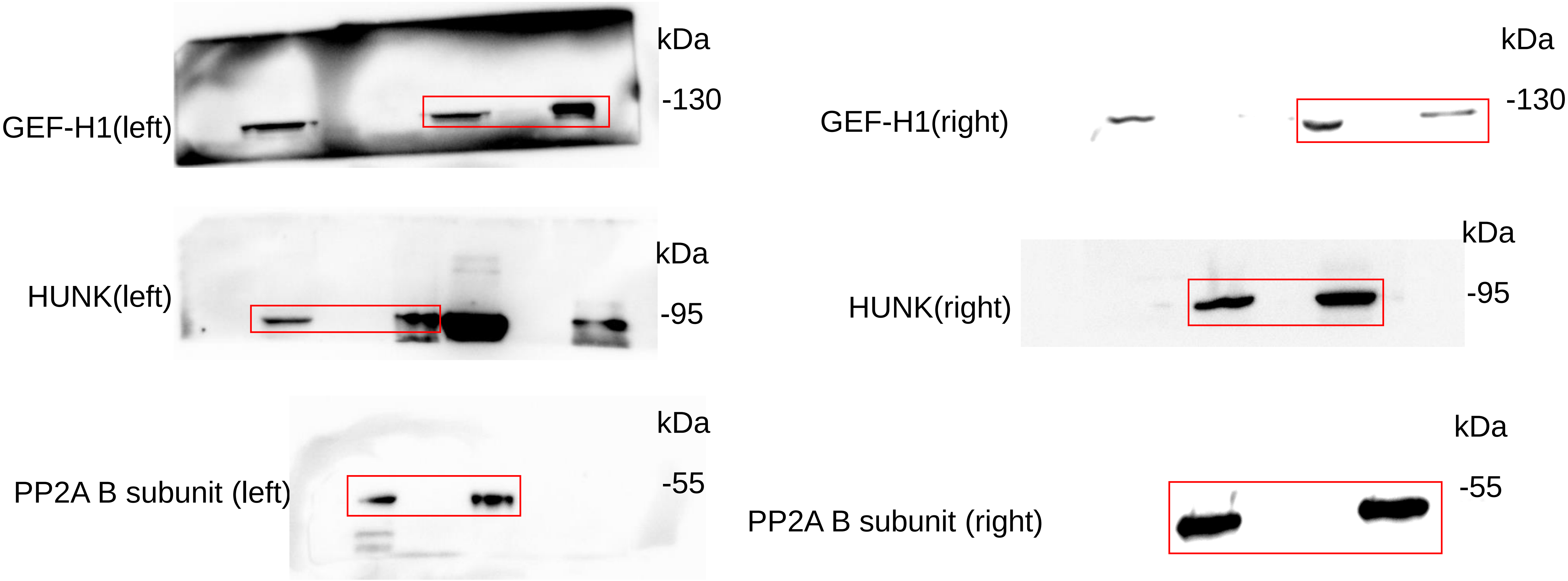
GEF-H1(left)
kDa
-130
GEF-H1(right)
kDa
-130
HUNK(left)
kDa
-95
HUNK(right)
kDa
-95
PP2A B subunit (left)
kDa
-55
PP2A B subunit (right)
kDa
-55

Fig 2E

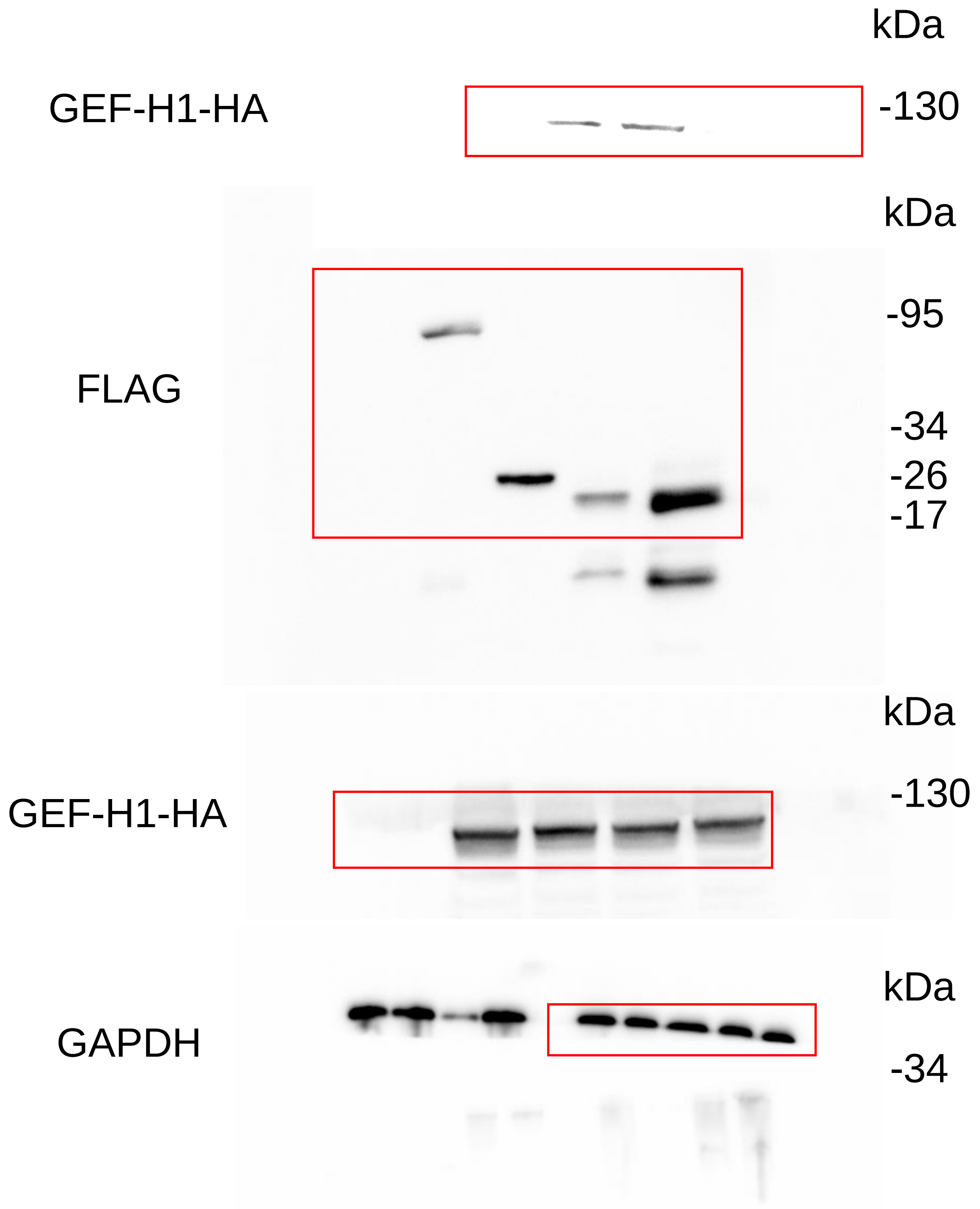
kDa
GEF-H1-HA
-130
kDa
-95
FLAG
-34
-26
-17
kDa
GEF-H1-HA
-130
kDa
GAPDH
-34

Fig 2F

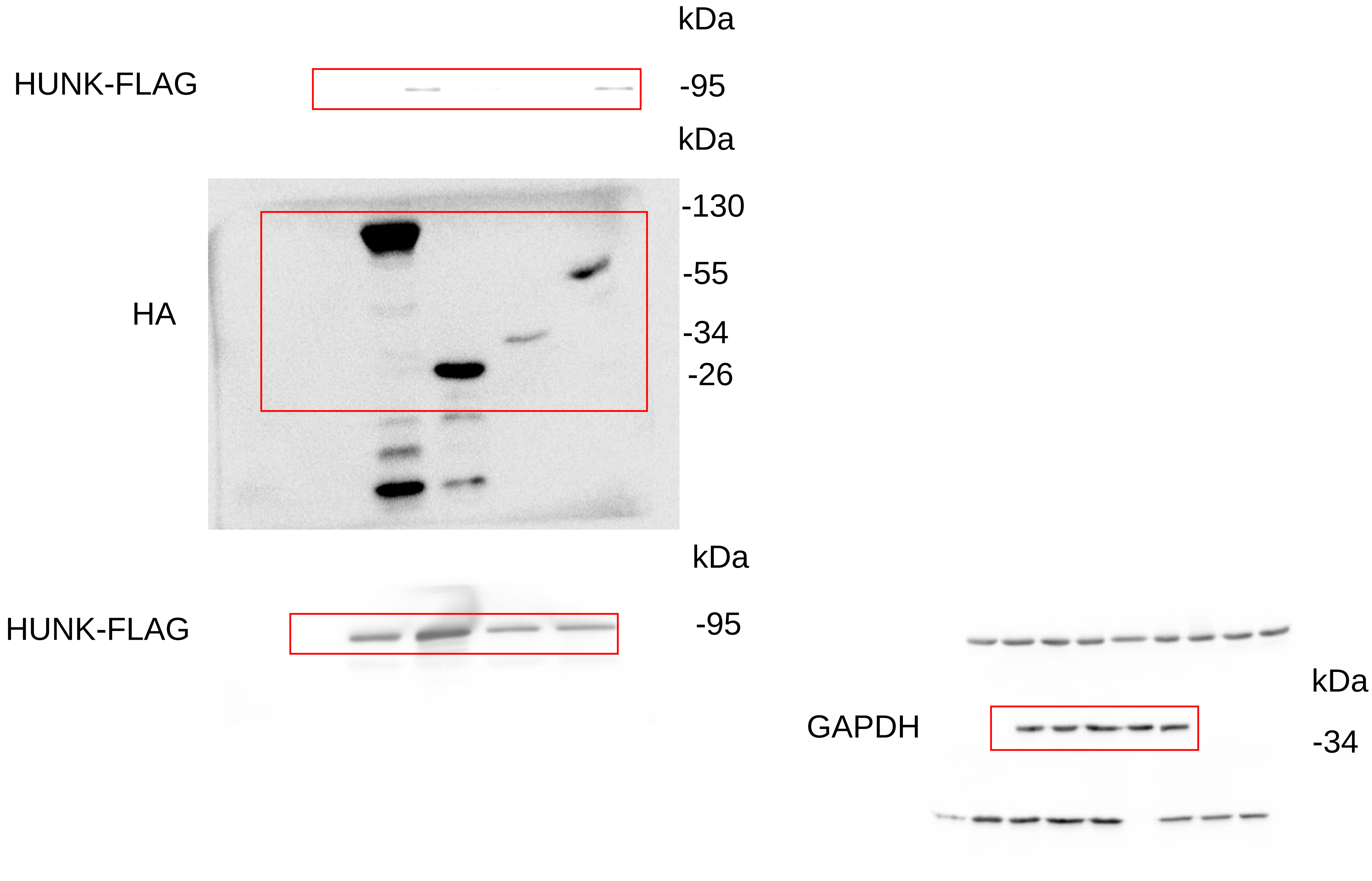
kDa
HUNK-FLAG
-95
kDa
-130
HA
-55
-34
-26
kDa
HUNK-FLAG
-95
kDa
GAPDH
-34

Fig 2G

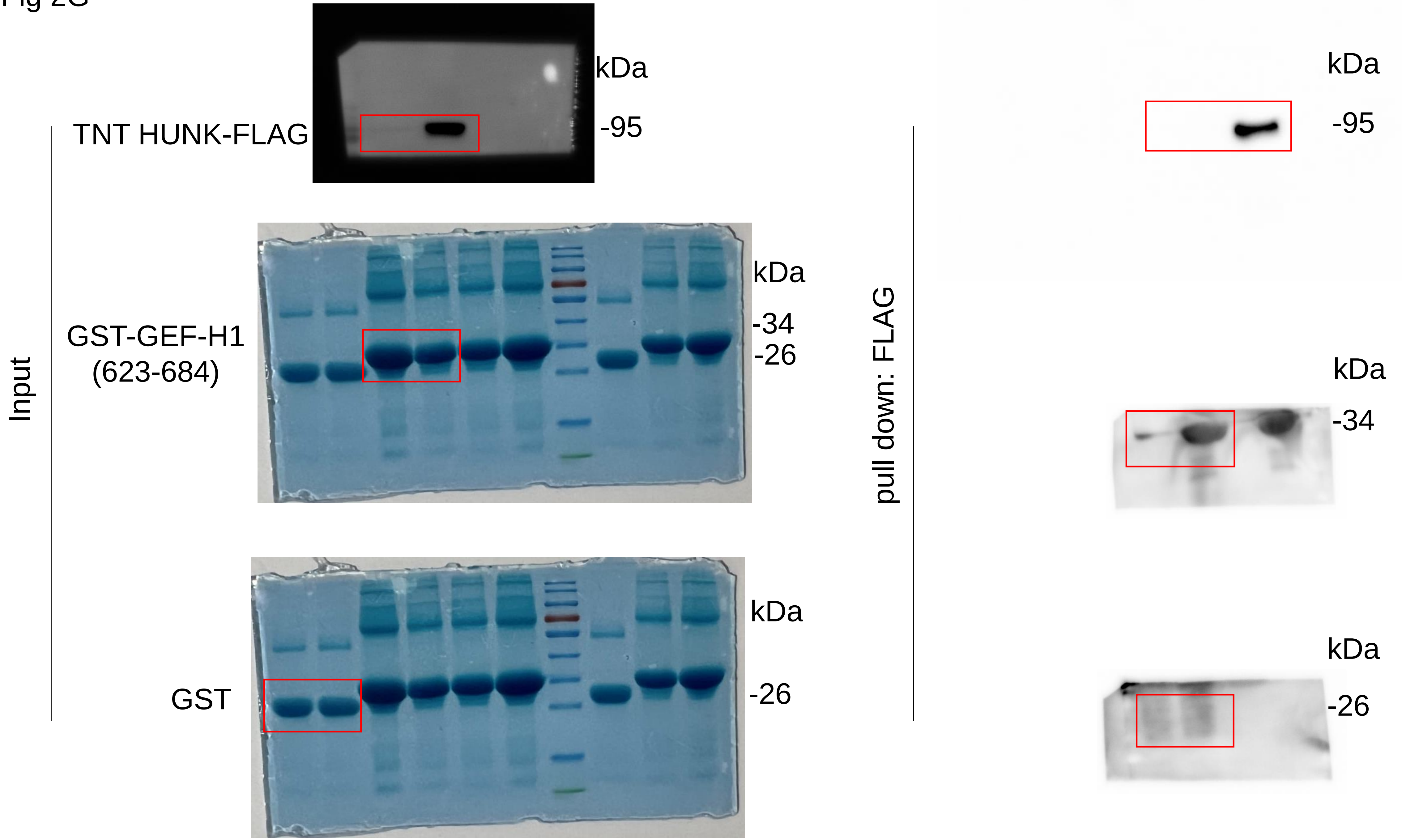

Input
TNT HUNK-FLAG
kDa
-95
GST-GEF-H1
(623-684)
kDa
-34
-26
GST
kDa
-26
pull down: FLAG
kDa
-95
kDa
-34
kDa
-26

Fig 2H

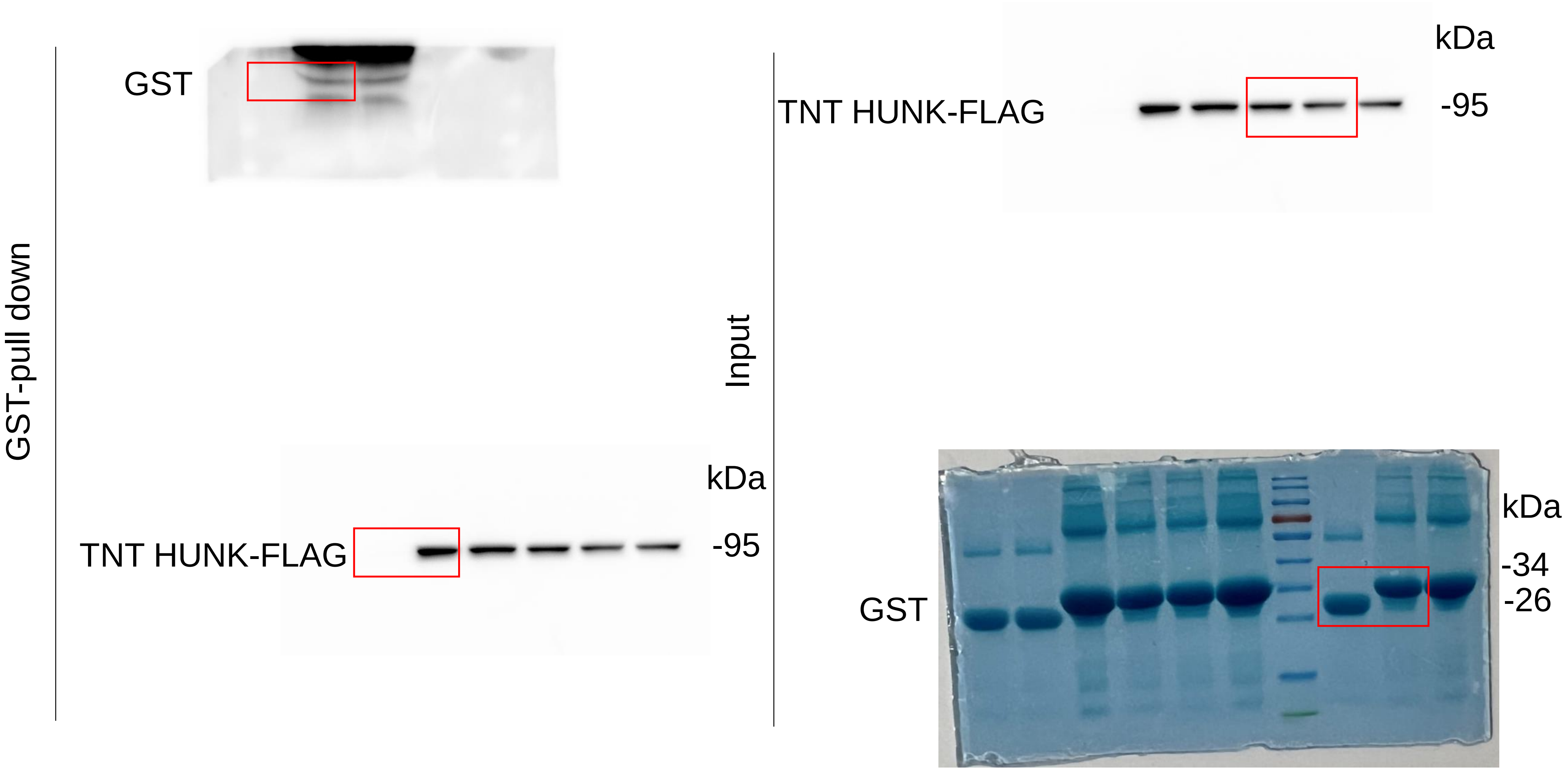
GST-pull down
GST
TNT HUNK-FLAG
kDa
-95
Input
TNT HUNK-FLAG
kDa
-95
GST
kDa
-34
-26

Fig 3D

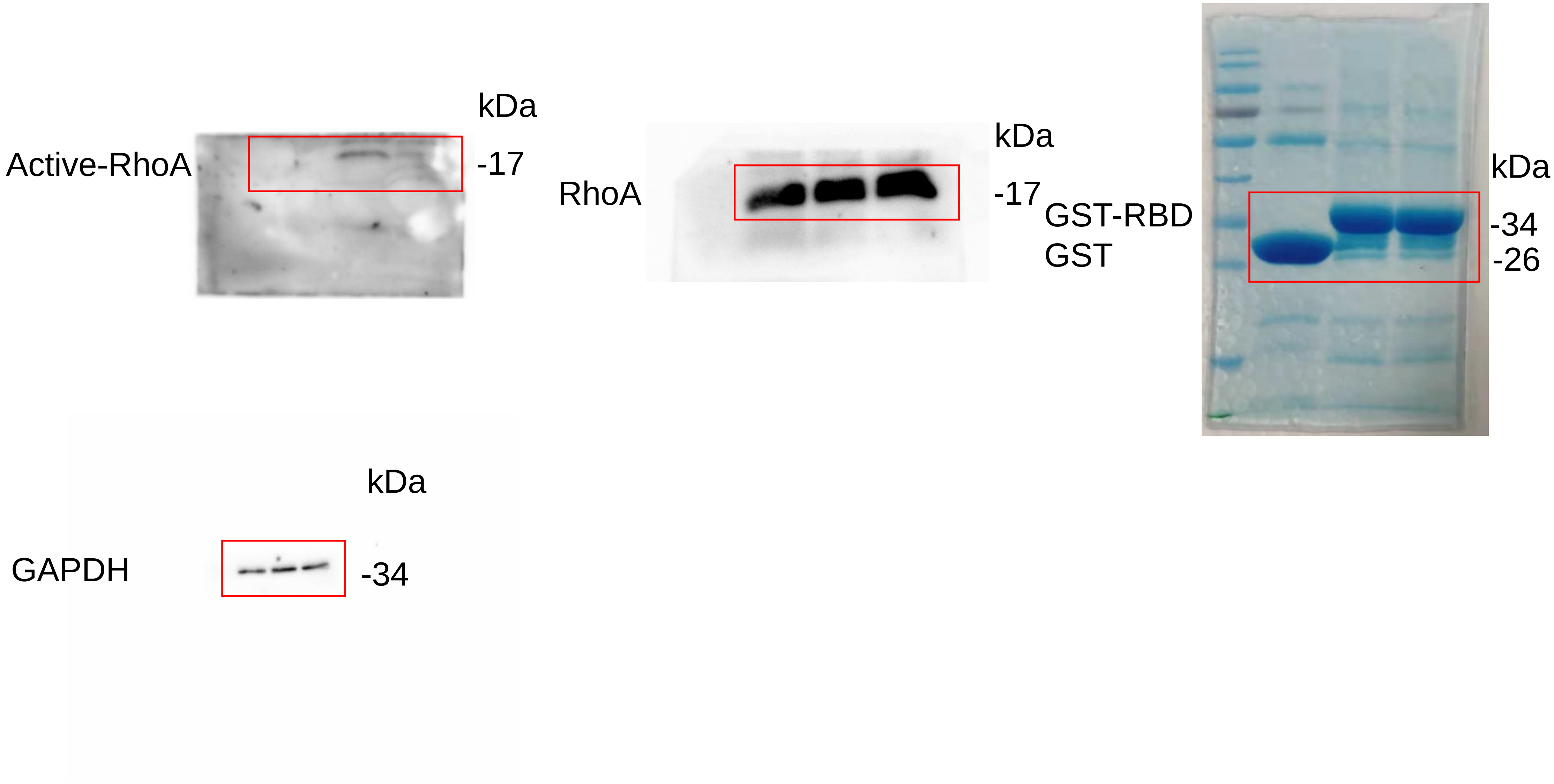
Active-RhoA
kDa
-17
RhoA
kDa
-17
GST-RBD
GST
kDa
-34
-26
GAPDH
kDa
-34

Fig 3E

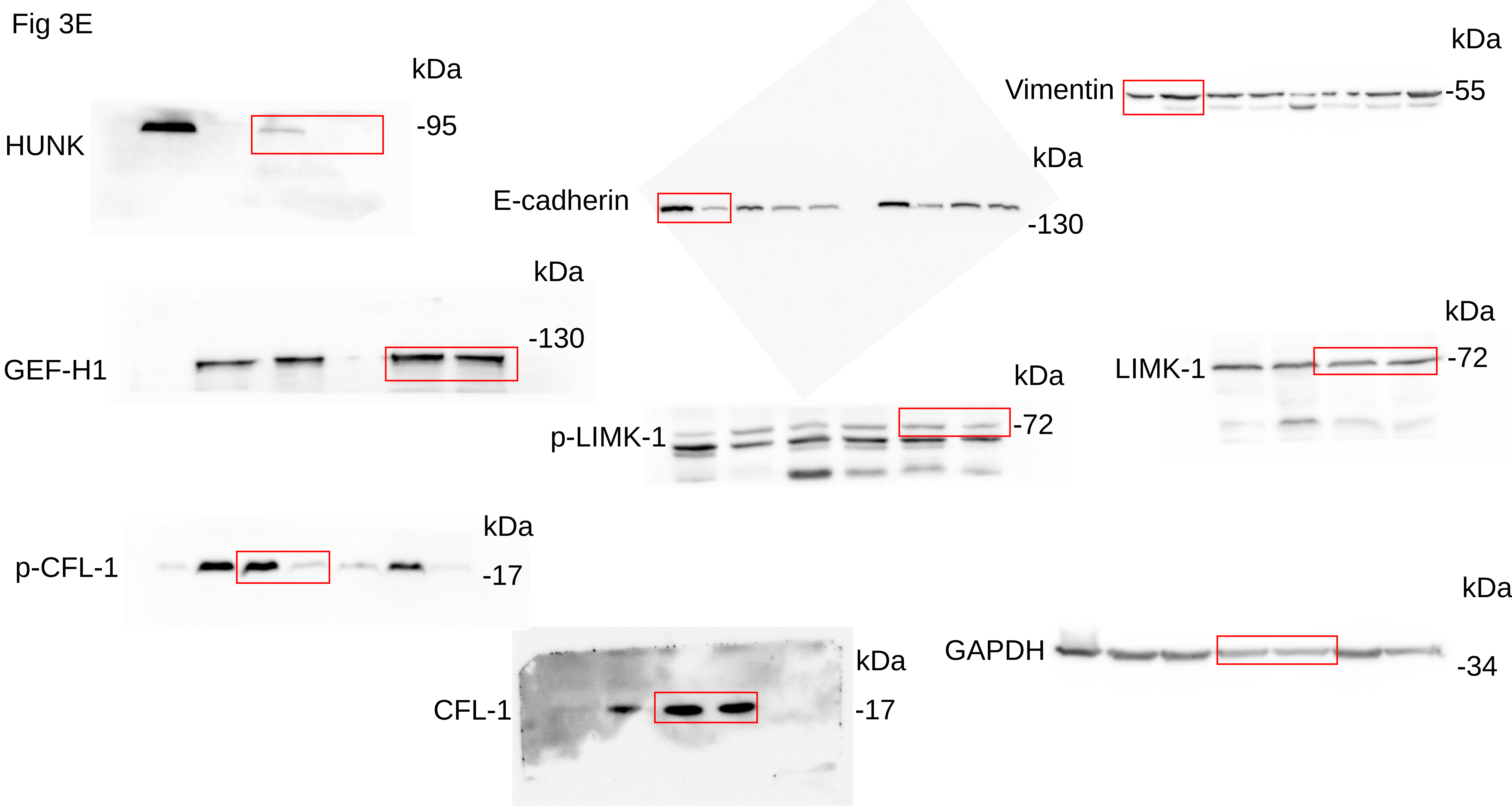
kDa
HUNK
-95
E-cadherin
kDa
-130
Vimentin
kDa
-55
GEF-H1
kDa
-130
p-LIMK-1
kDa
-72
LIMK-1
kDa
-72
p-CFL-1
kDa
-17
CFL-1
kDa
-17
GAPDH
kDa
-34

Fig 3F

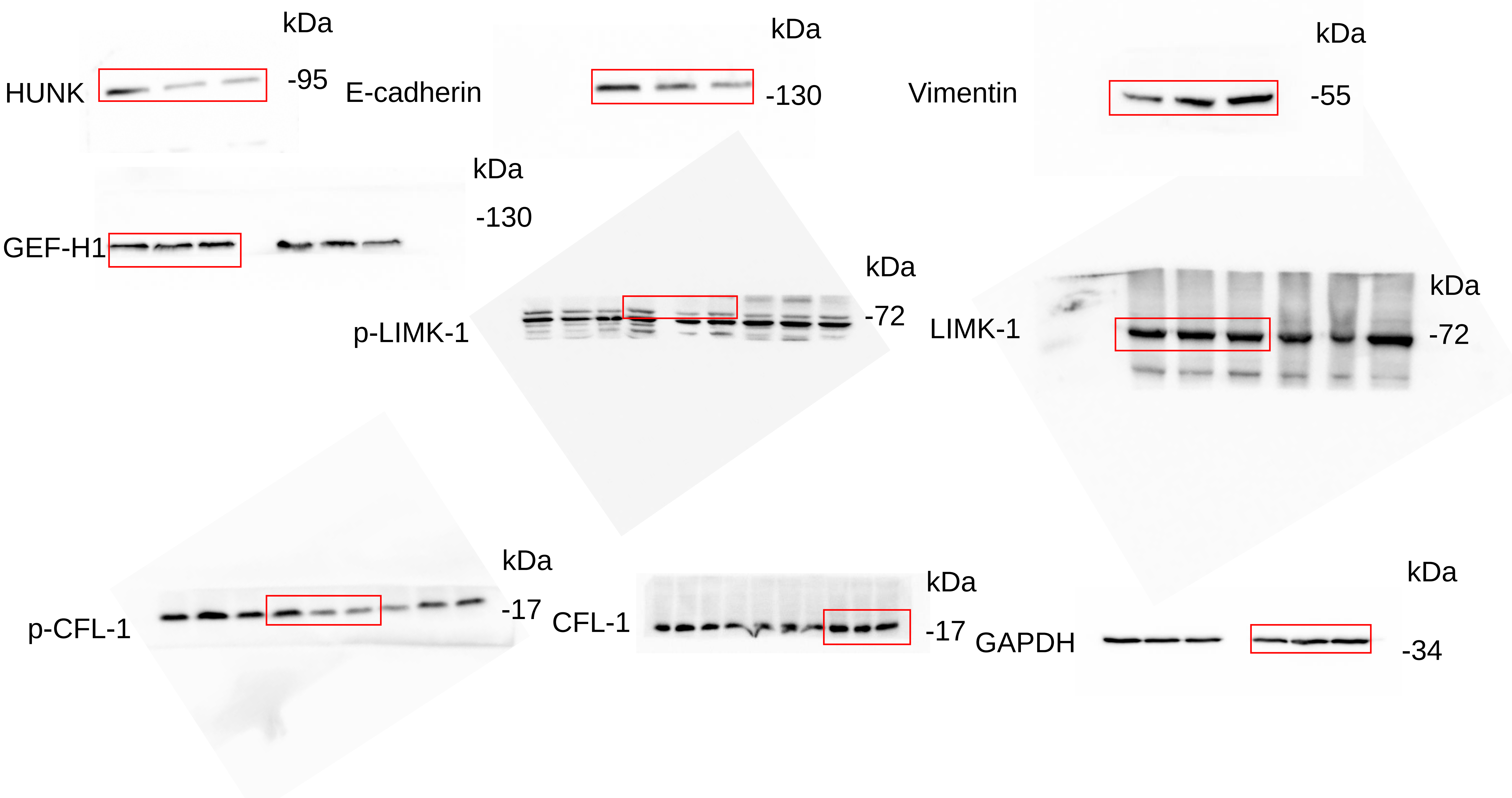
kDa
HUNK
-95
kDa
E-cadherin
-130
kDa
Vimentin
-55
kDa
-130
GEF-H1
kDa
-72
p-LIMK-1
kDa
LIMK-1
-72
kDa
-17
p-CFL-1
kDa
CFL-1
-17
kDa
GAPDH
-34

Fig 3G

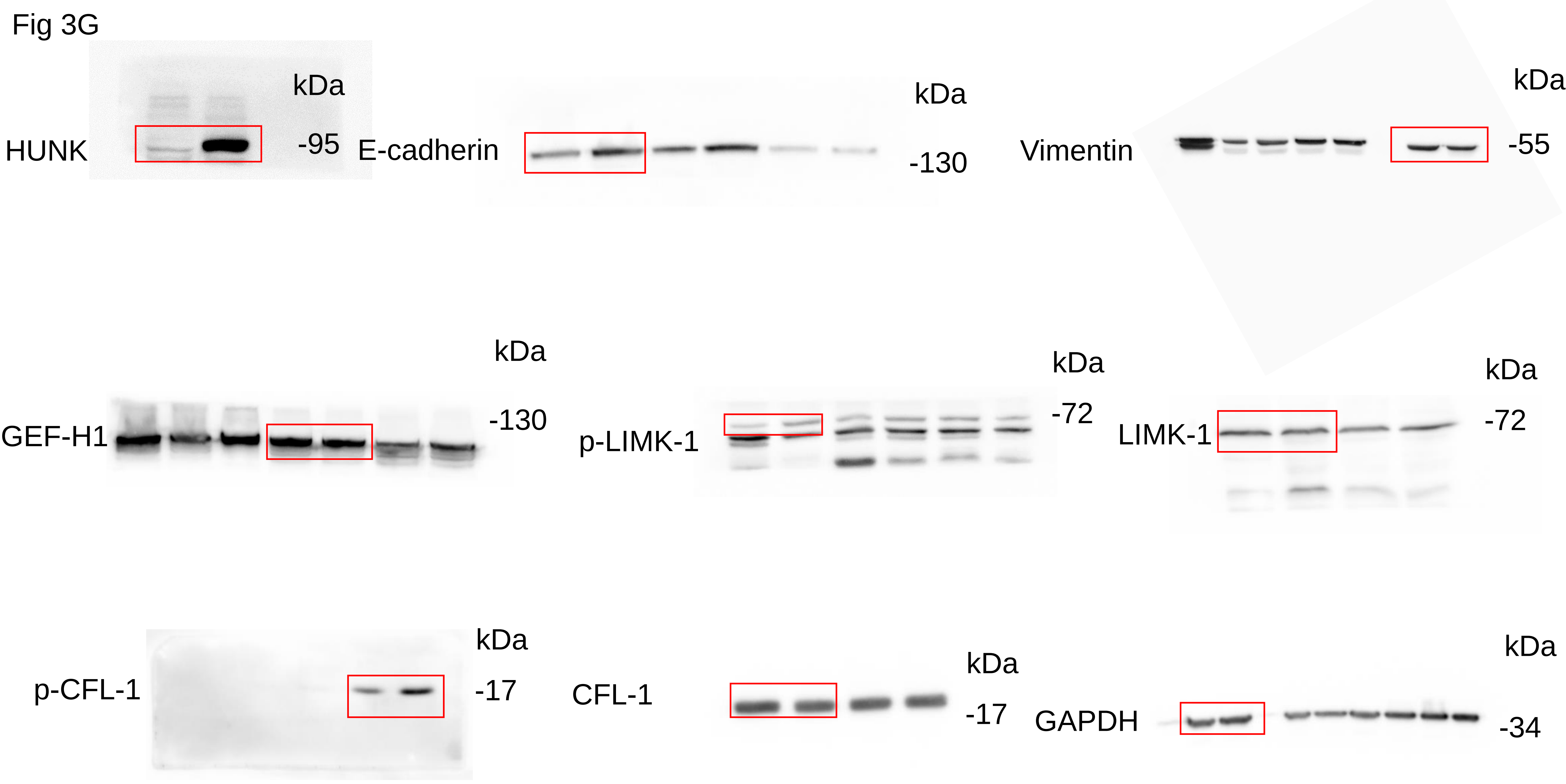
HUNK
kDa
-95
E-cadherin
kDa
-130
Vimentin
kDa
-55
GEF-H1
kDa
-130
p-LIMK-1
kDa
-72
LIMK-1
kDa
-72
p-CFL-1
kDa
-17
CFL-1
kDa
-17
GAPDH
kDa
-34

Fig 3I

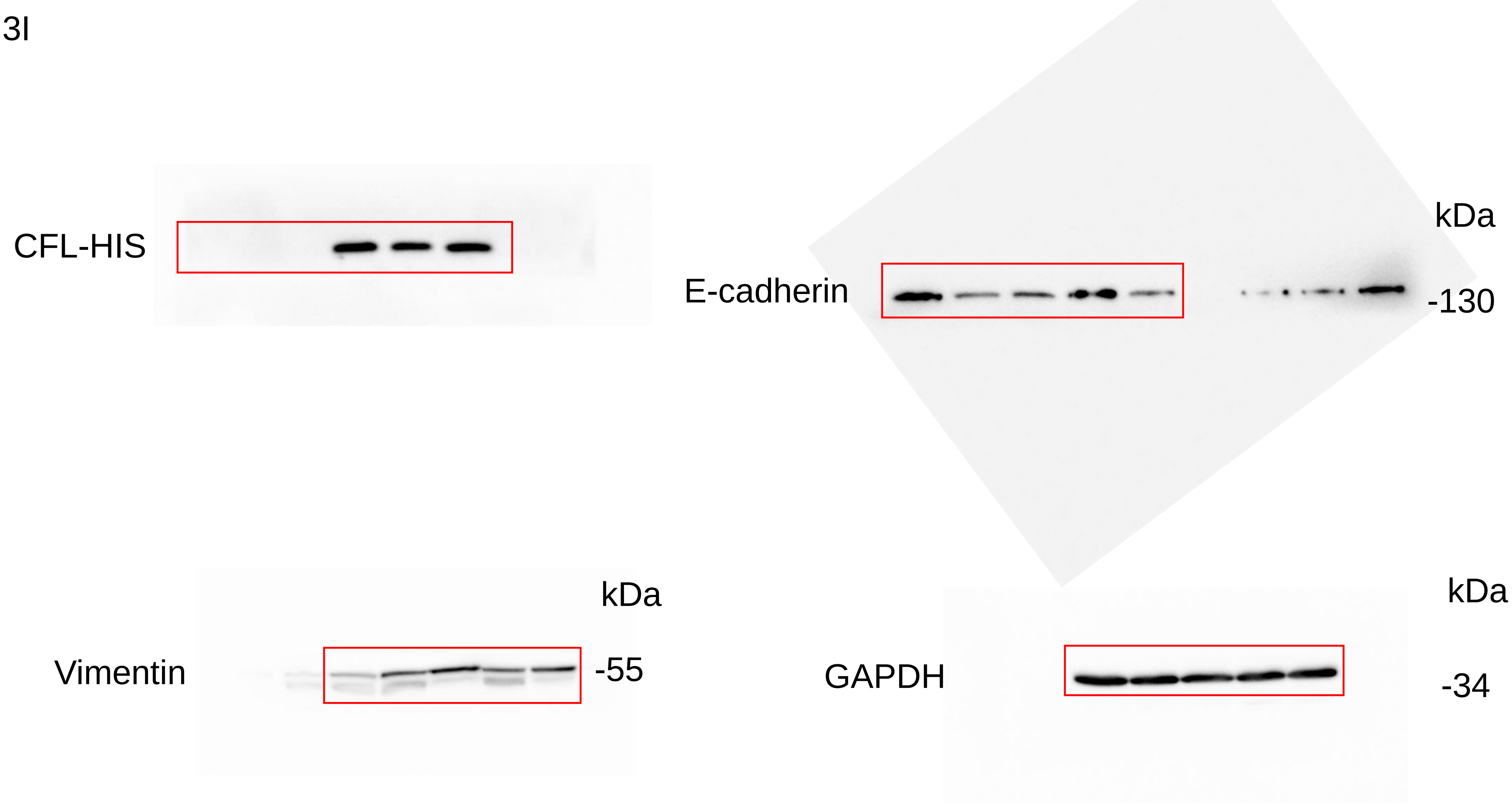

Fig 4B

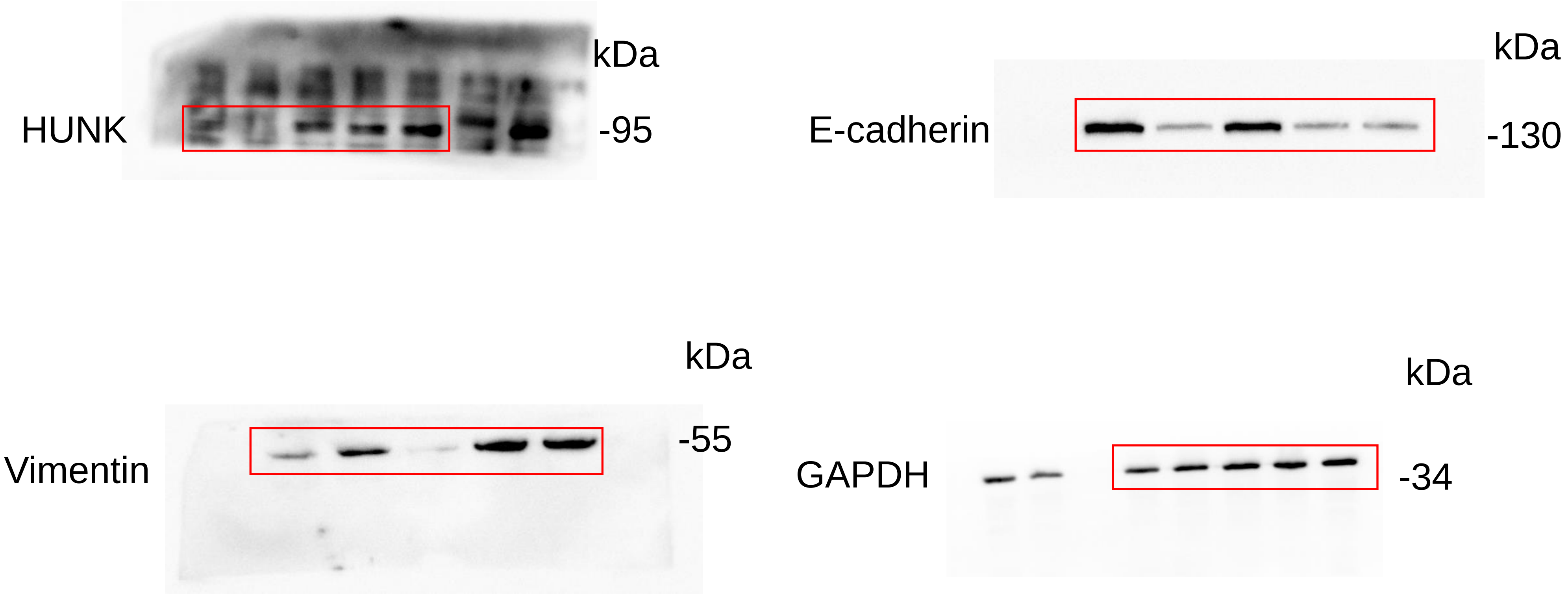

Fig 4H

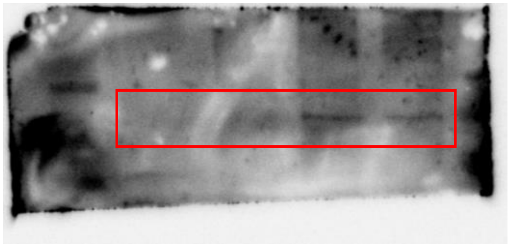

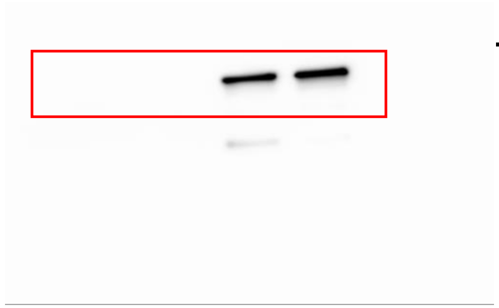

Fig 4I

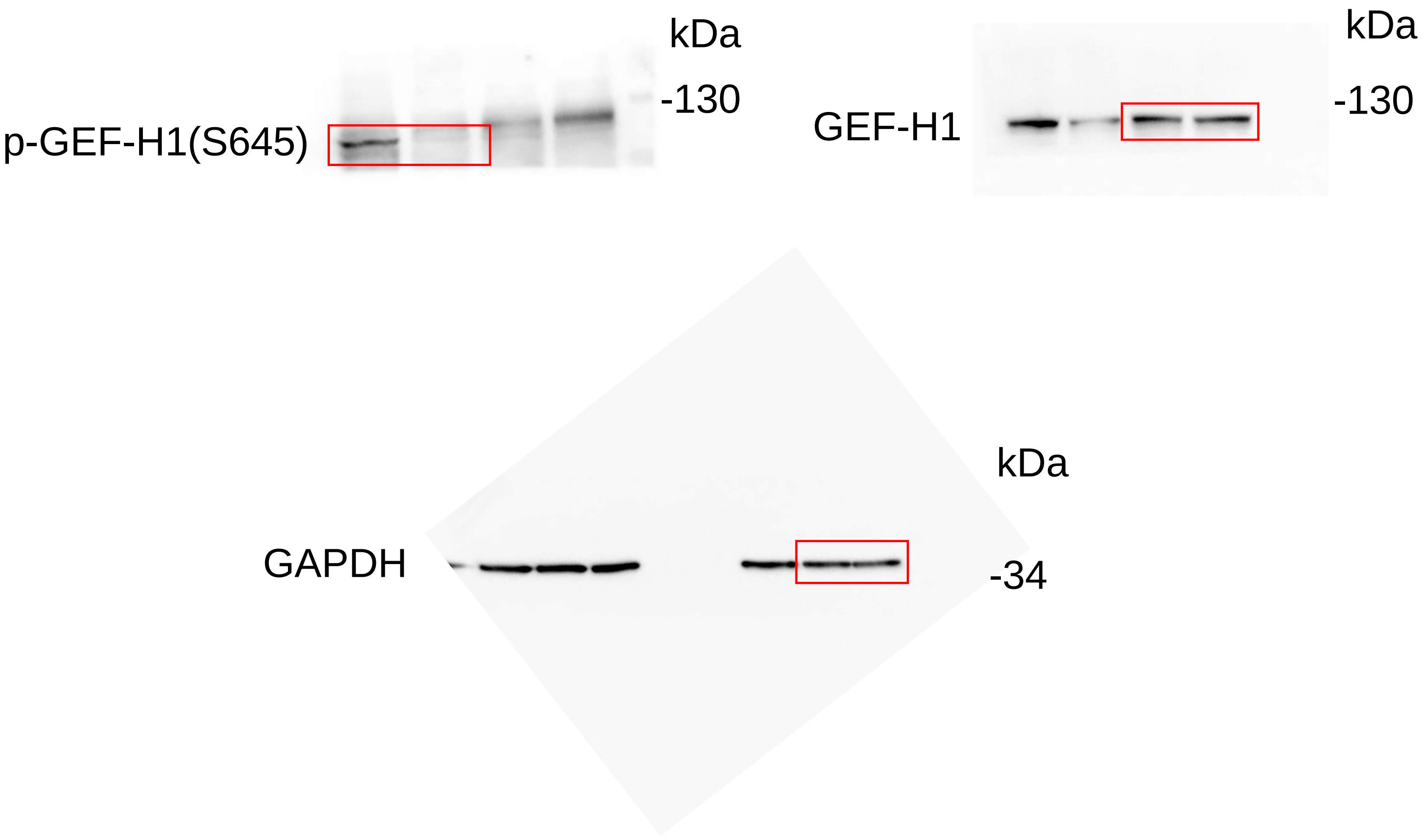

Fig 4K

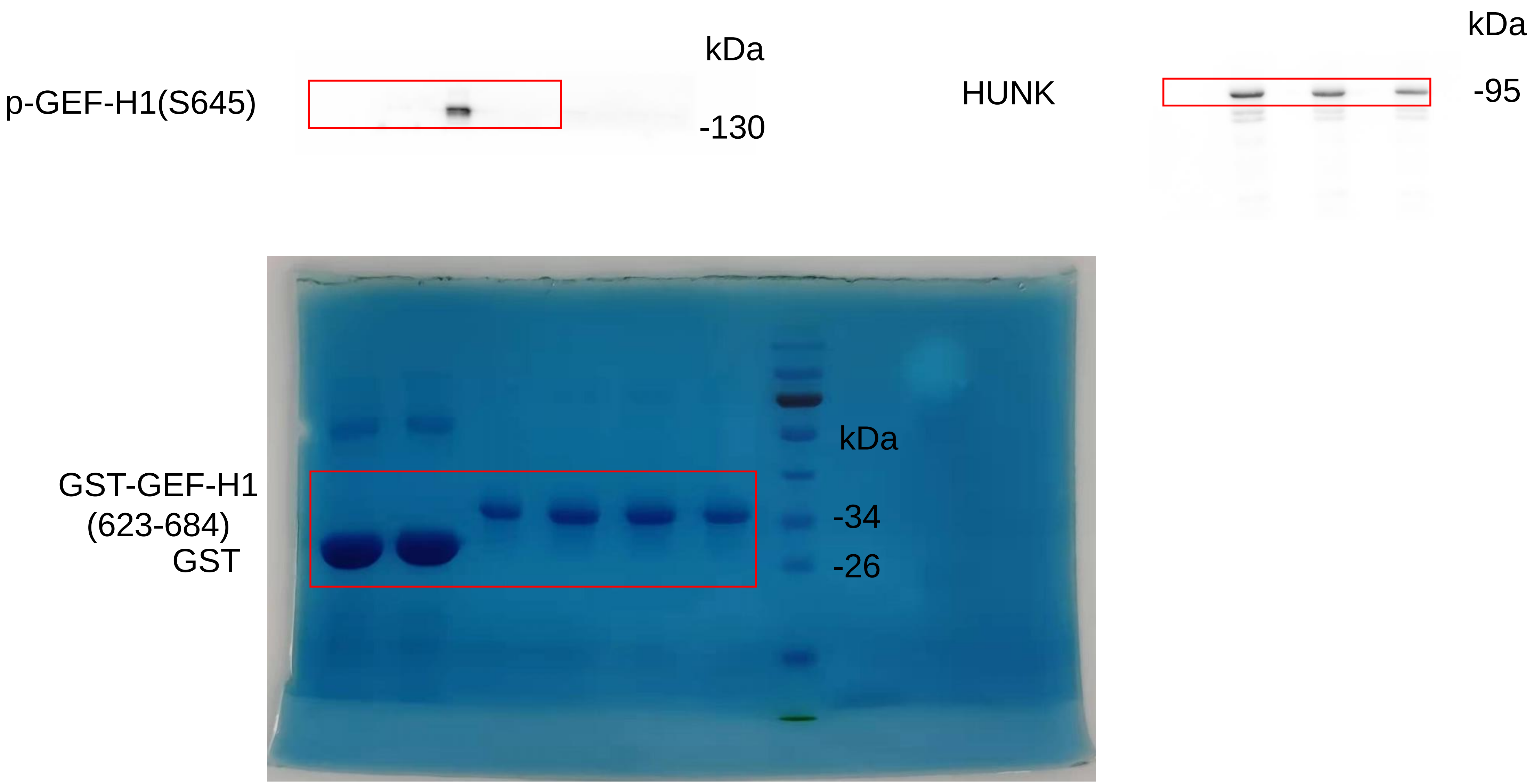

Fig 4L

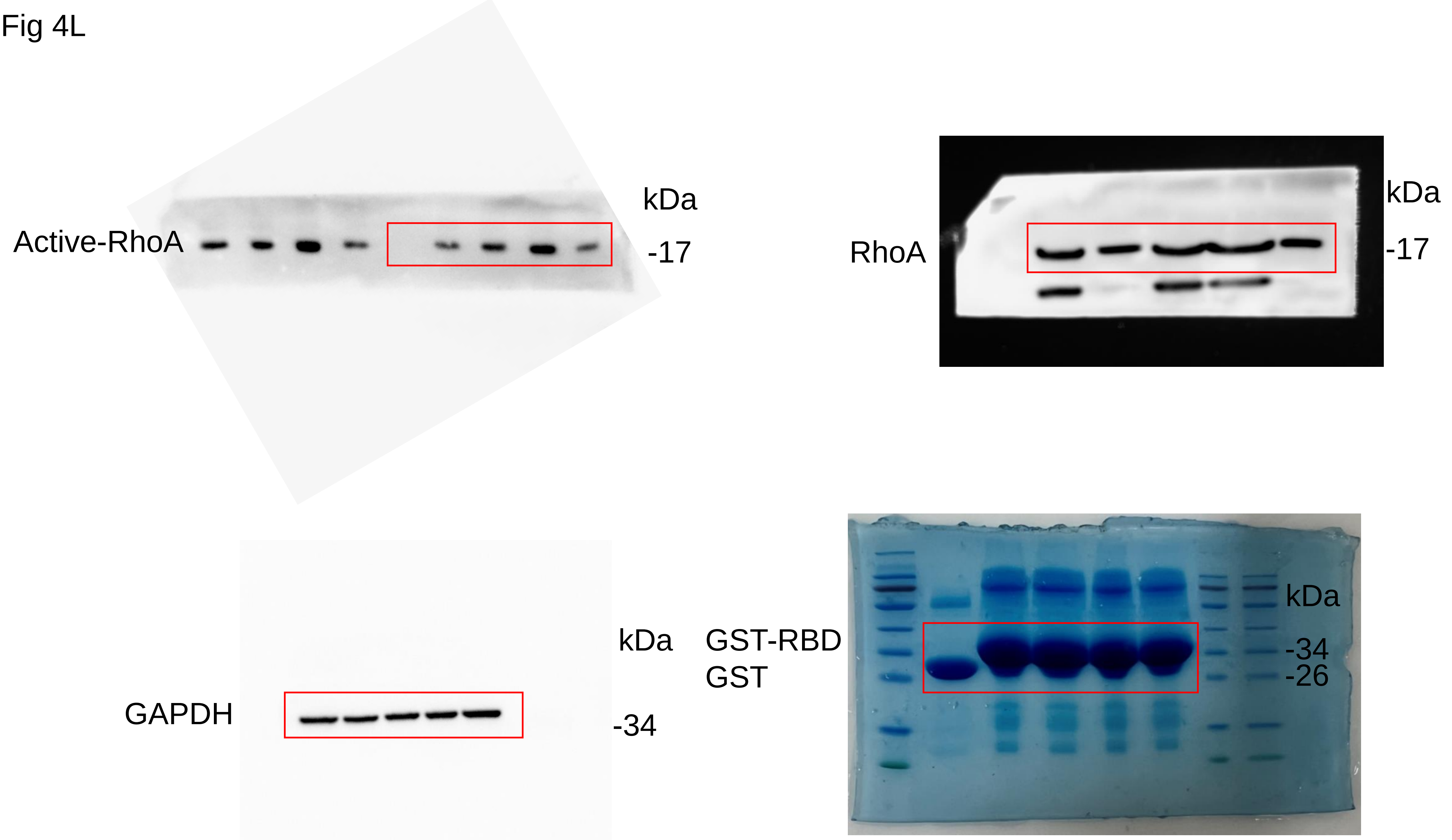
Active-RhoA
kDa
-17
RhoA
kDa
-17
GAPDH
kDa
-34
GST-RBD
GST
kDa
-34
-26

Fig 4J

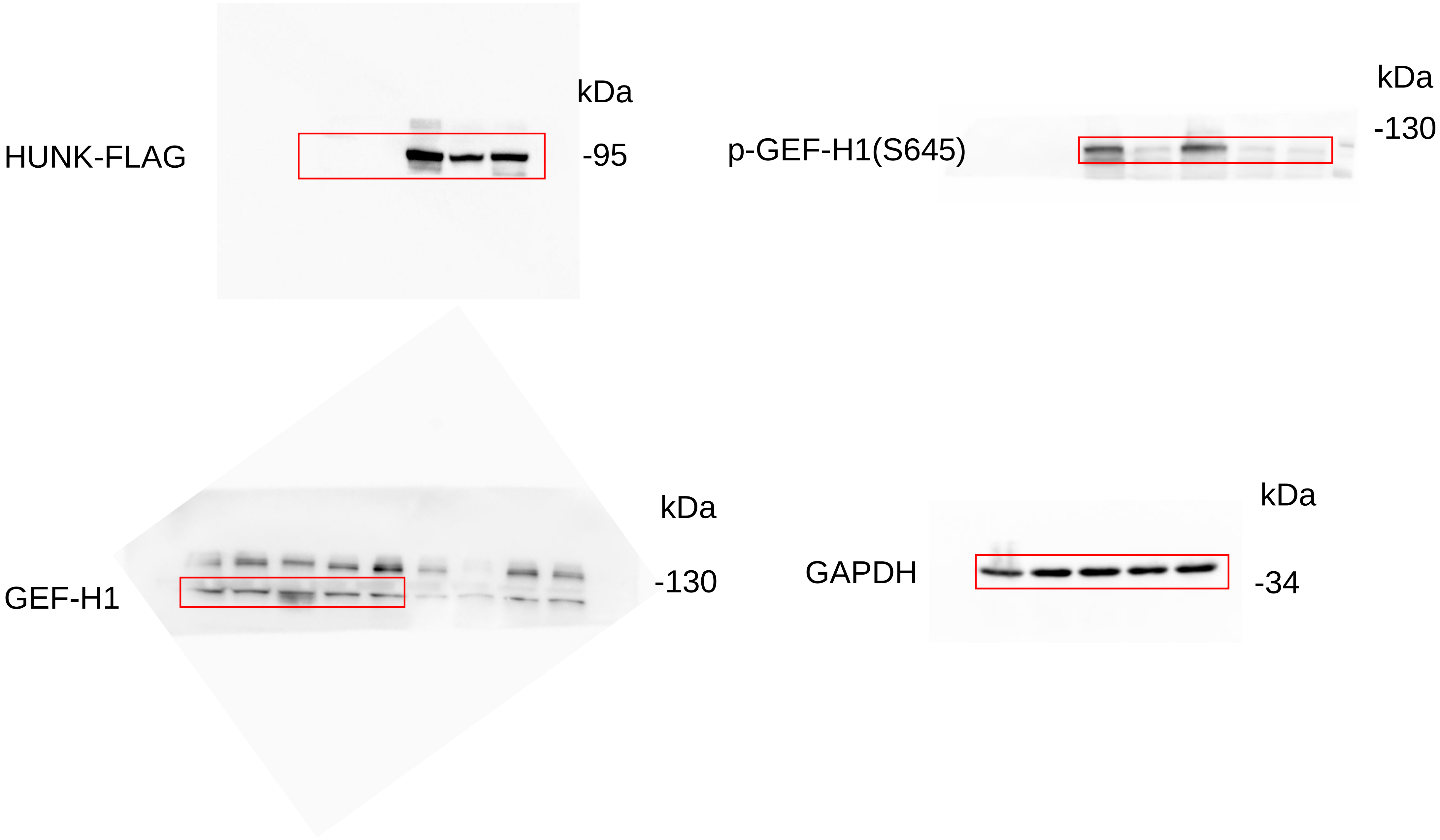
HUNK-FLAG
kDa
-95
p-GEF-H1(S645)
kDa
-130
GEF-H1
kDa
-130
GAPDH
kDa
-34

Fig 4N

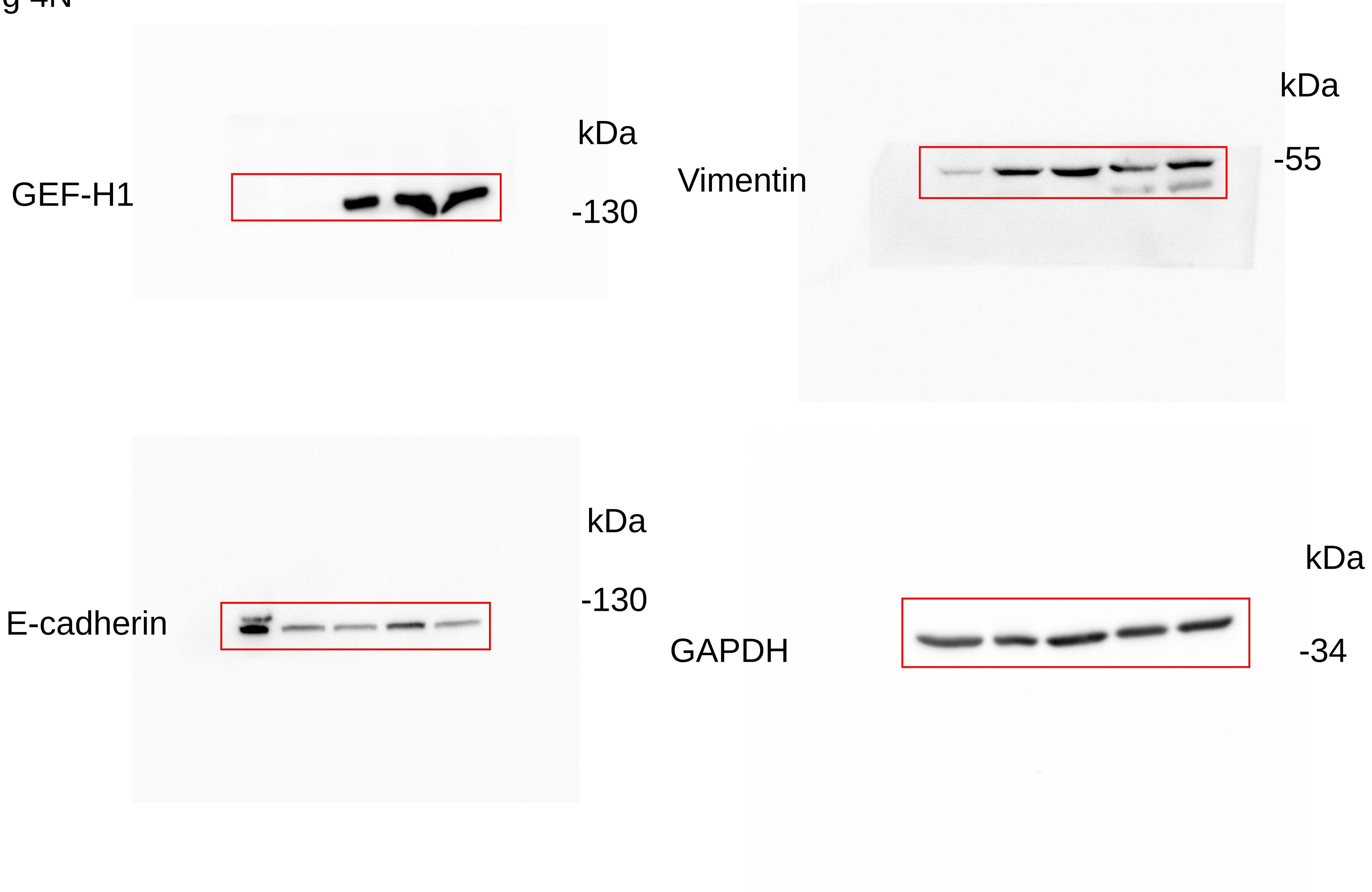

Fig 5B

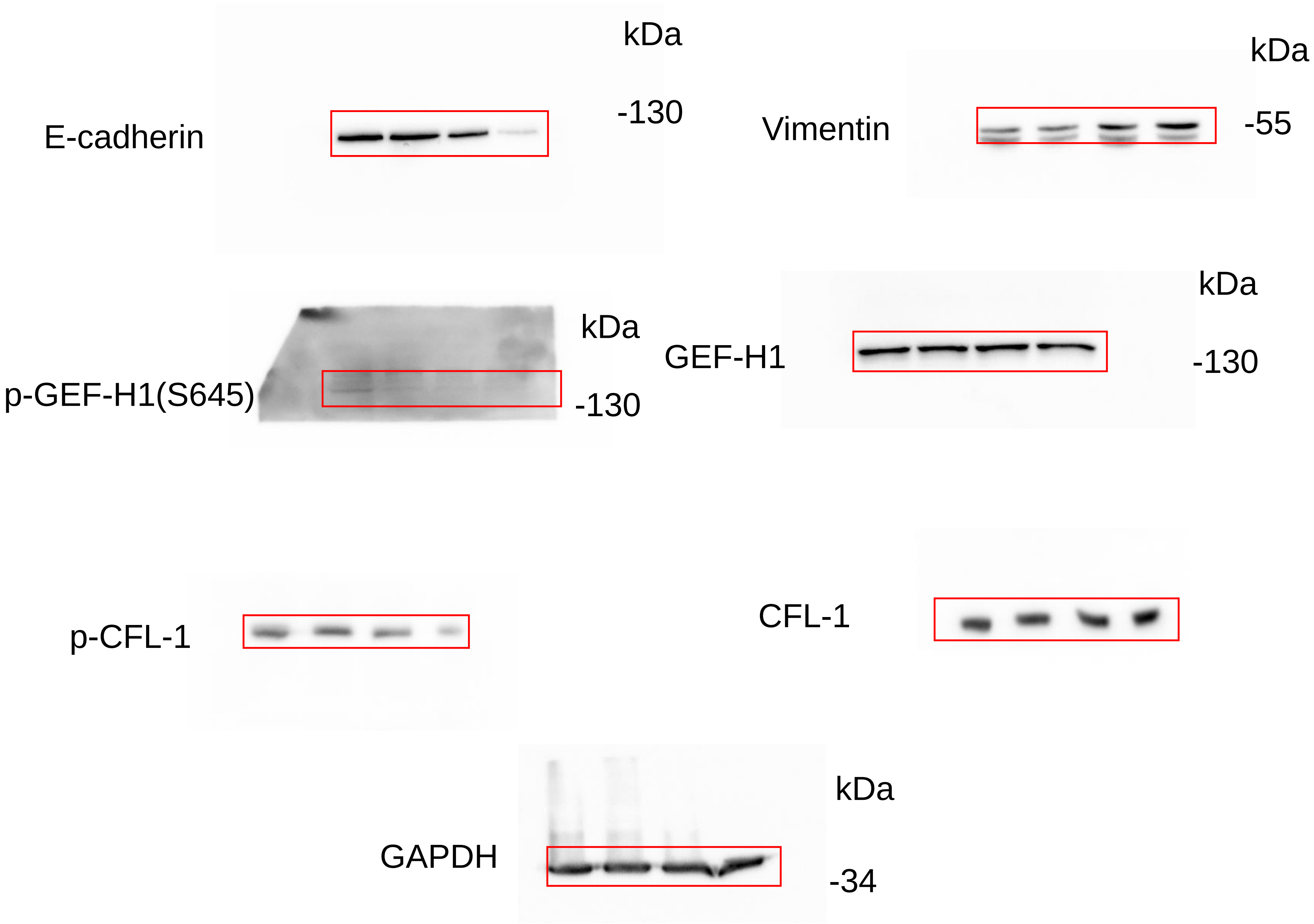
E-cadherin
kDa
-130
Vimentin
kDa
-55
p-GEF-H1(S645)
kDa
-130
GEF-H1
kDa
-130
p-CFL-1
CFL-1
GAPDH
kDa
-34

Fig S1F

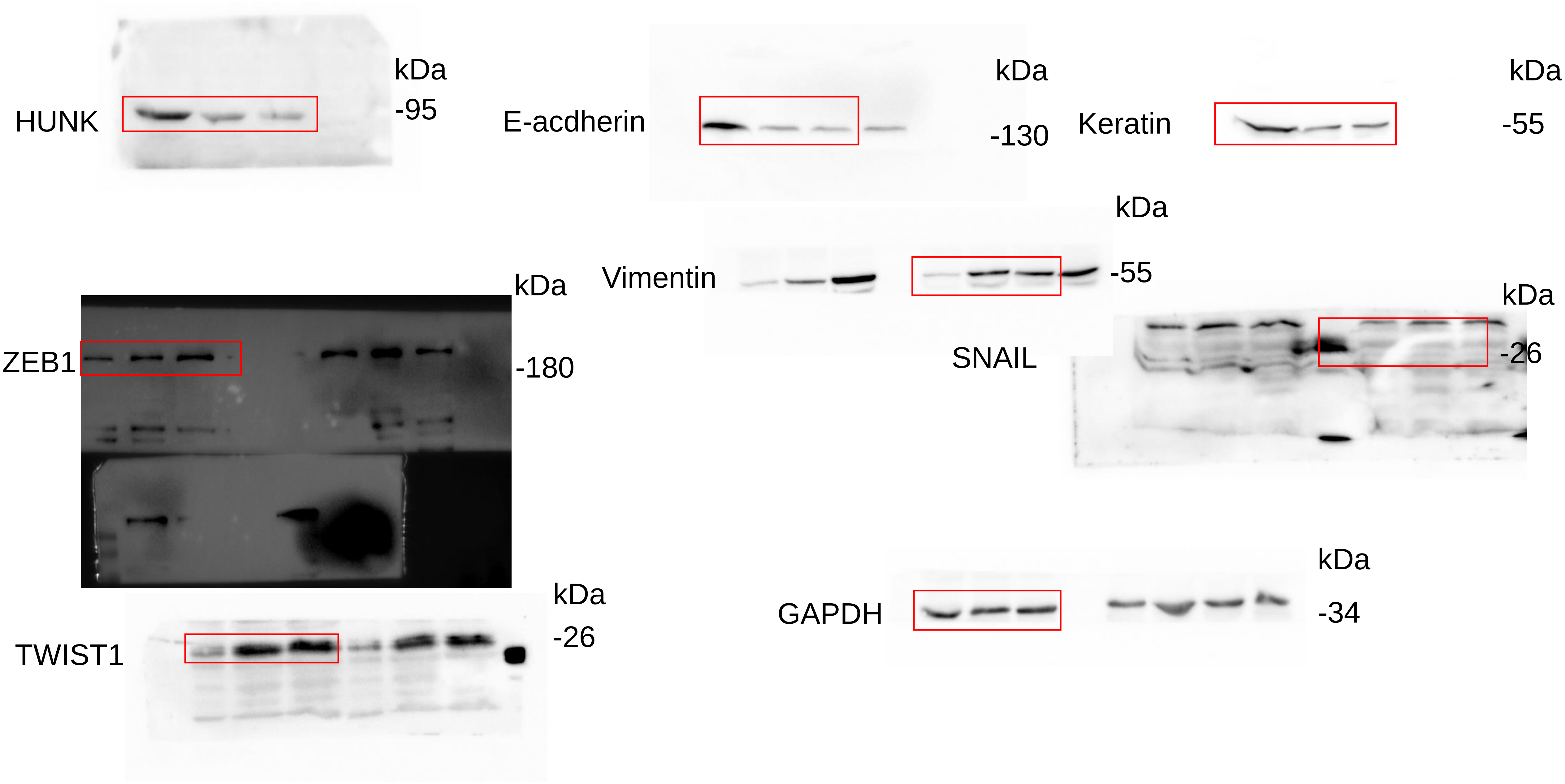
HUNK
kDa
-95
E-acdherin
kDa
-130
Keratin
kDa
-55
Vimentin
kDa
-55
ZEB1
kDa
-180
SNAIL
kDa
-26
TWIST1
kDa
-26
GAPDH
kDa
-34

Fig S1H

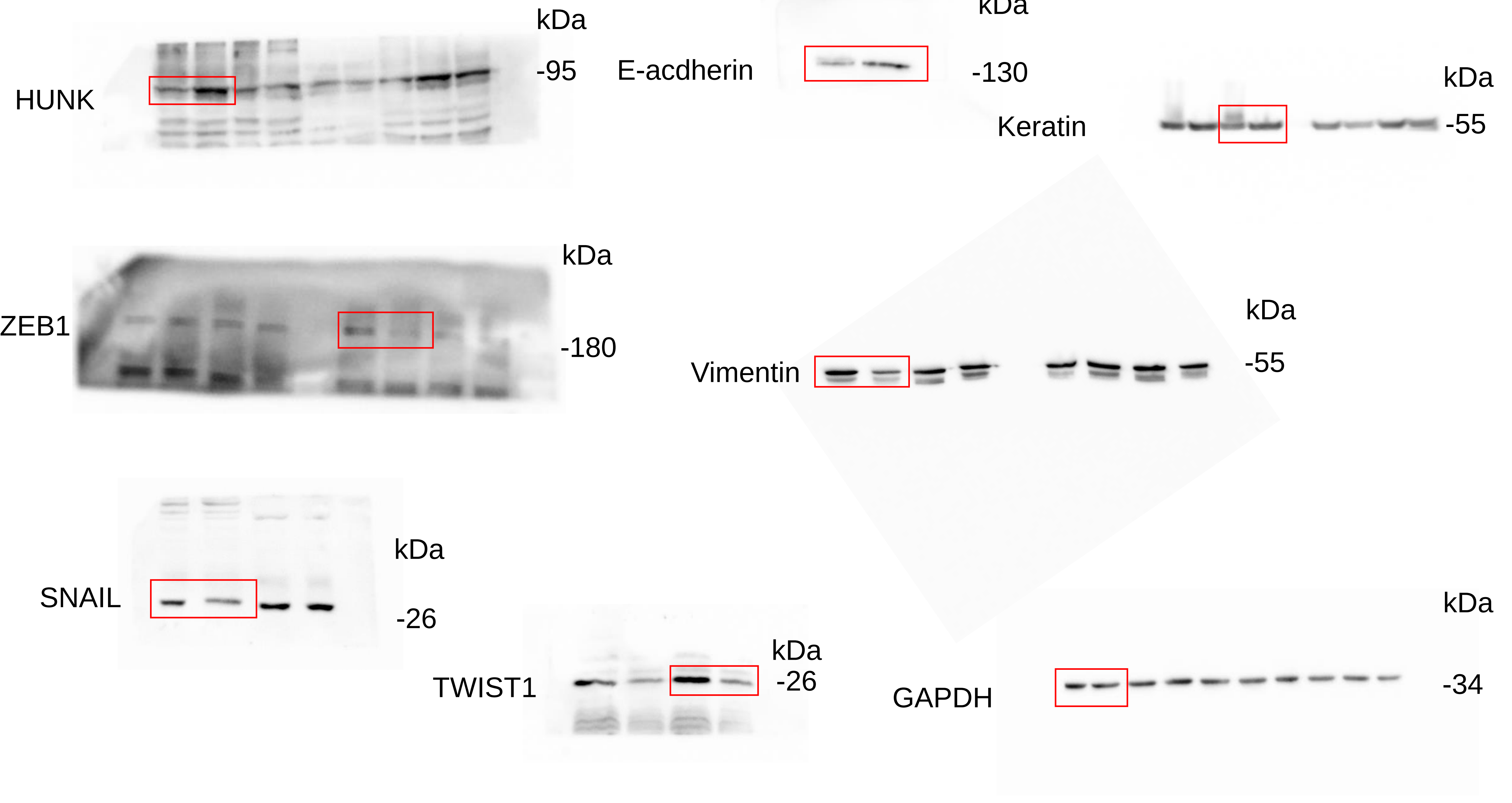
HUNK
kDa
-95
E-acdherin
kDa
-130
Keratin
kDa
-55
ZEB1
kDa
-180
Vimentin
kDa
-55
SNAIL
kDa
-26
TWIST1
kDa
-26
GAPDH
kDa
-34

Fig S2C

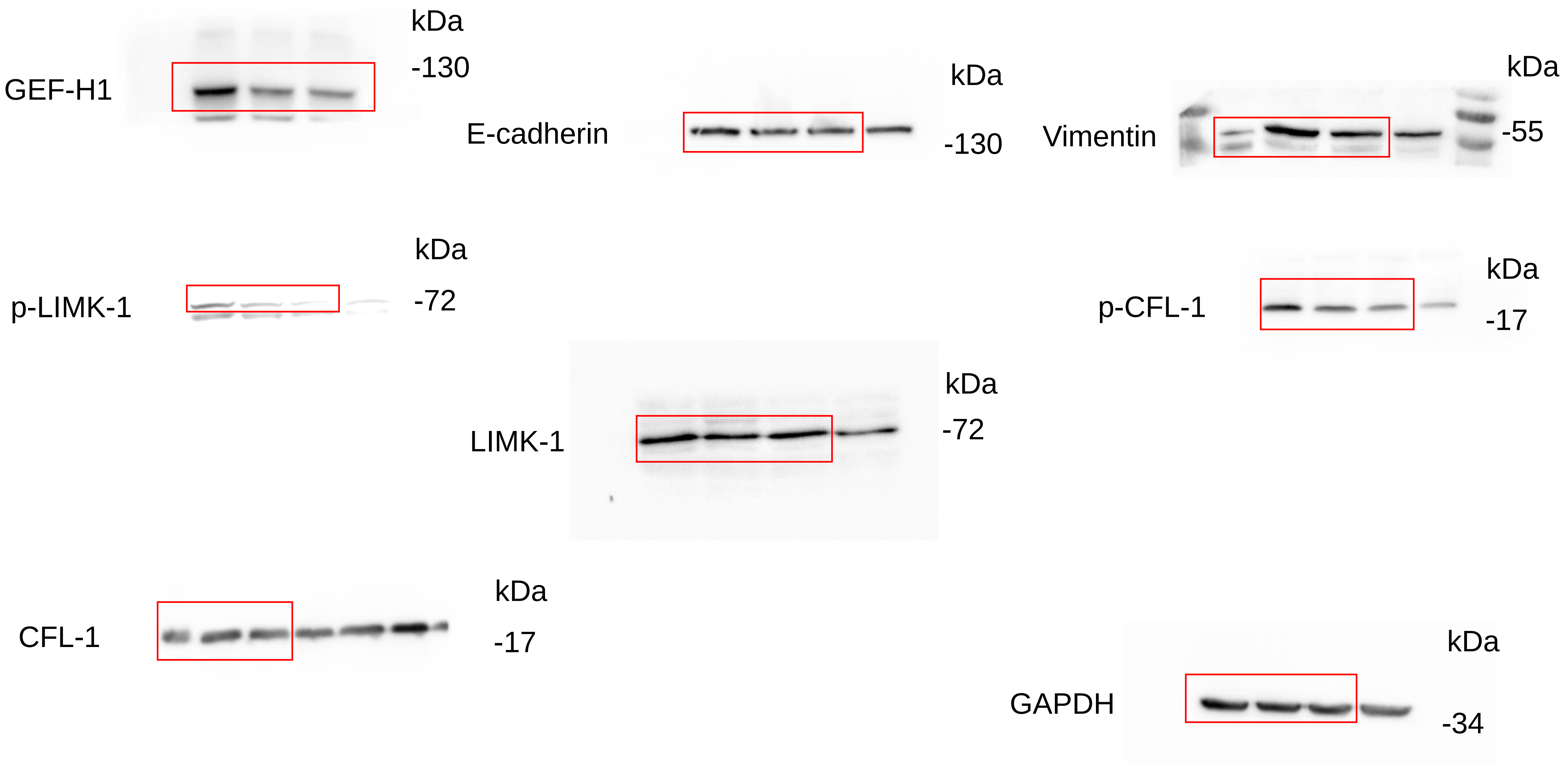
GEF-H1
kDa
-130
E-cadherin
kDa
-130
Vimentin
kDa
-55
p-LIMK-1
kDa
-72
p-CFL-1
kDa
-17
LIMK-1
kDa
-72
CFL-1
kDa
-17
GAPDH
kDa
-34

Fig S3B

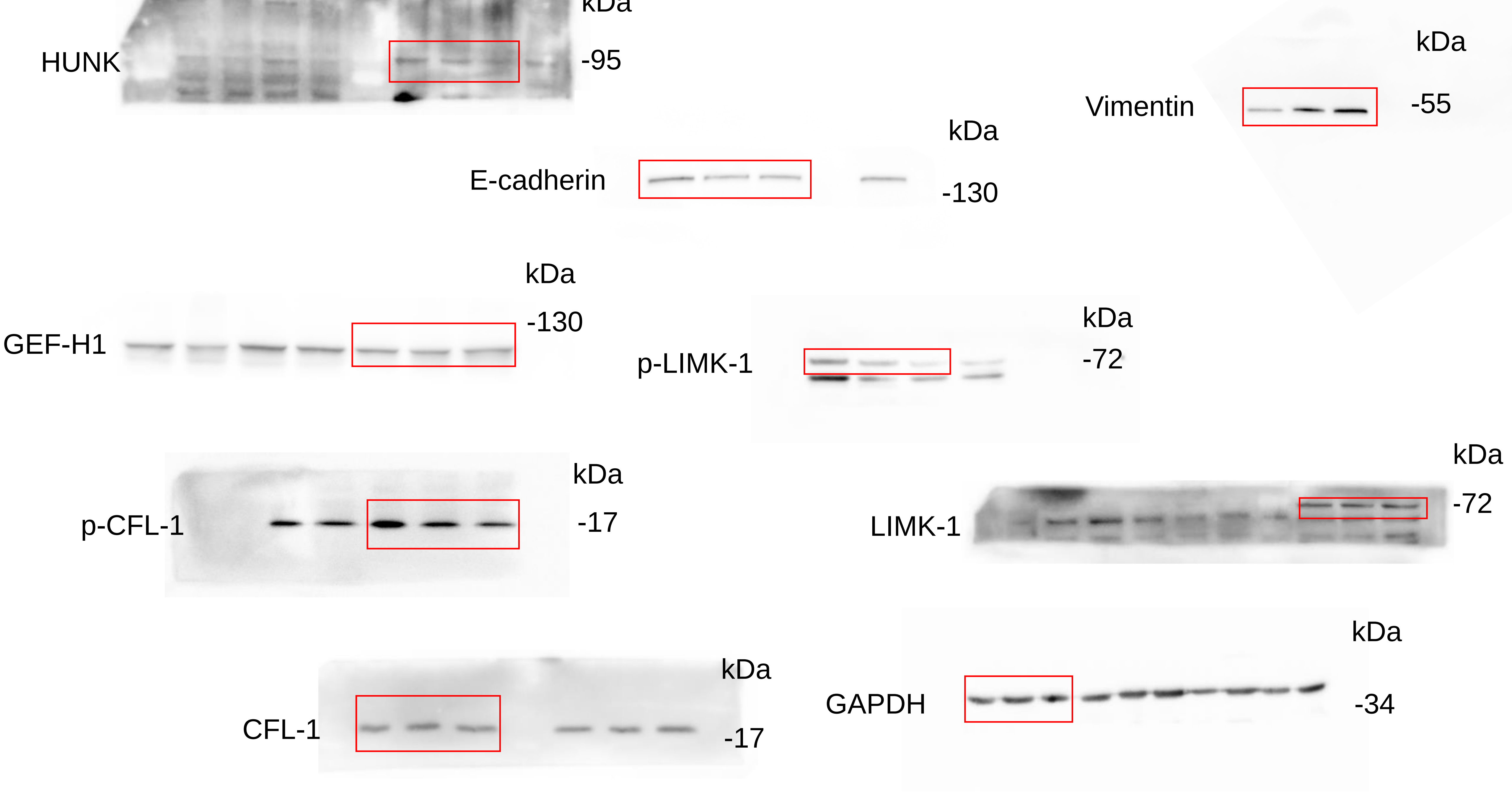
HUNK
kDa
-95
Vimentin
kDa
-55
E-cadherin
kDa
-130
GEF-H1
kDa
-130
p-LIMK-1
kDa
-72
p-CFL-1
kDa
-17
LIMK-1
kDa
-72
CFL-1
kDa
-17
GAPDH
kDa
-34

Fig S3C

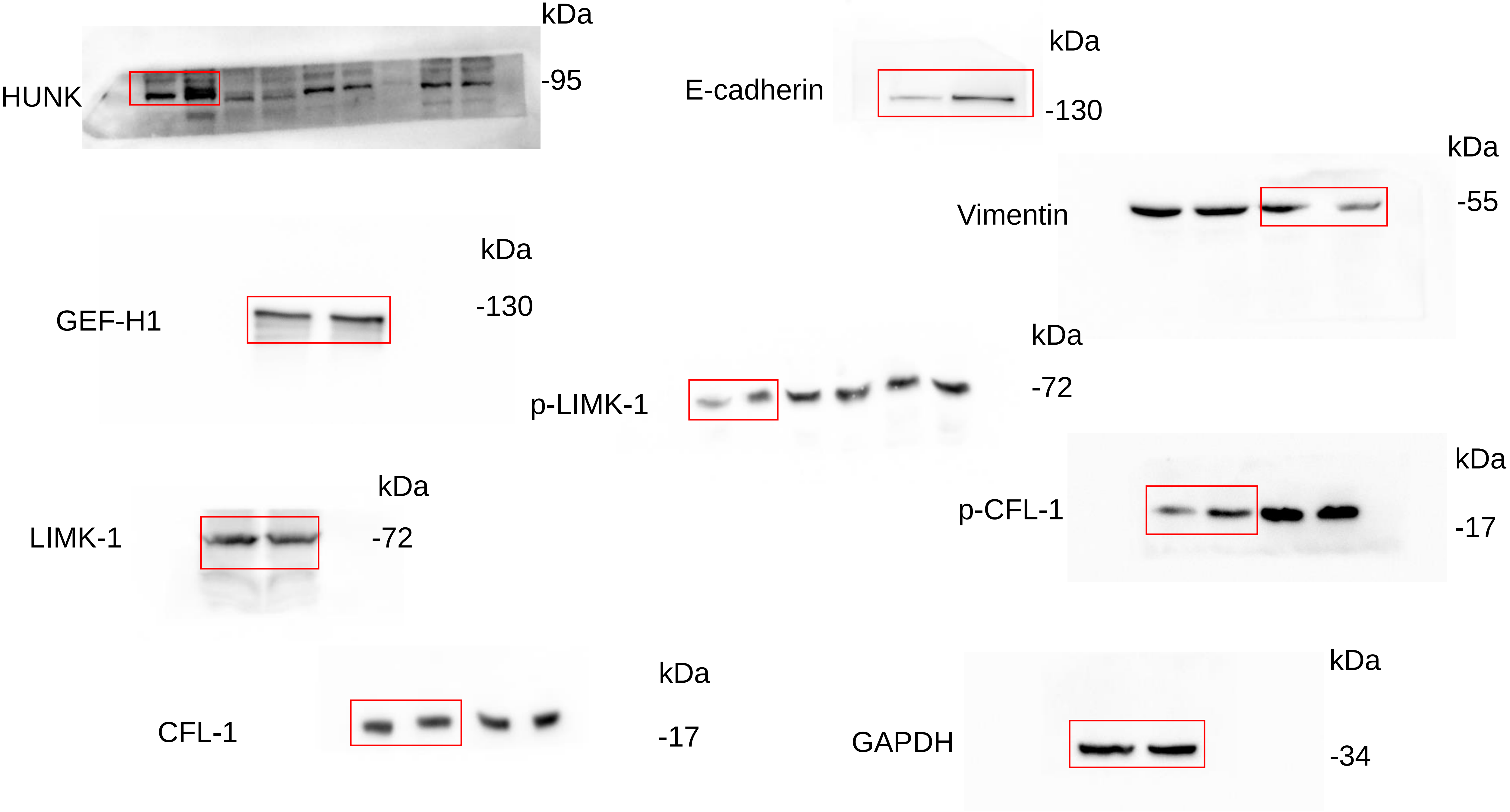

HUNK
kDa
-95
E-cadherin
kDa
-130
kDa
-55
Vimentin
kDa
GEF-H1
-130
kDa
p-LIMK-1
-72
LIMK-1
kDa
-72
kDa
p-CFL-1
-17
CFL-1
kDa
-17
kDa
GAPDH
-34

Fig S3E

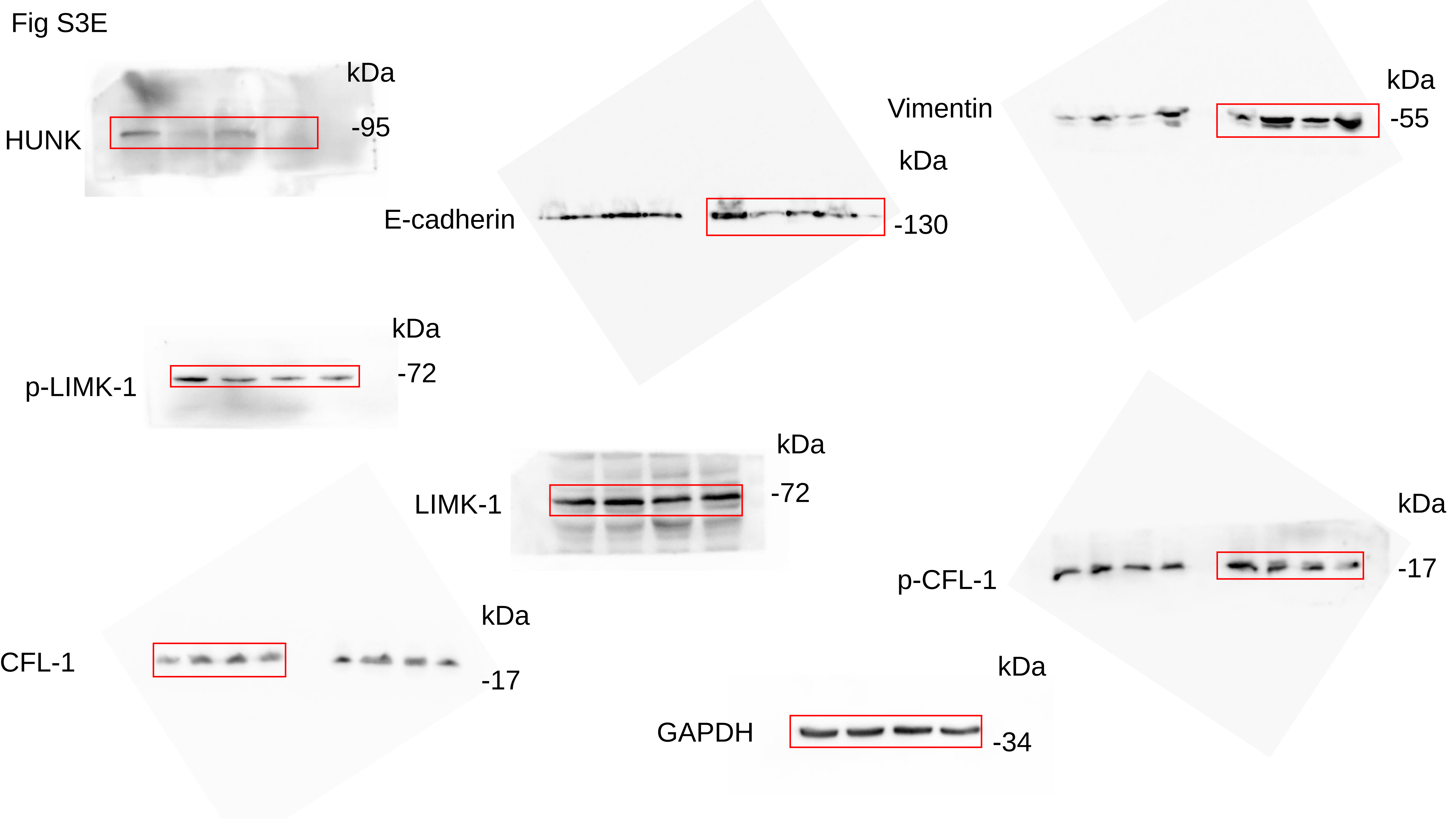
kDa
HUNK
-95
E-cadherin
kDa
-130
Vimentin
kDa
-55
p-LIMK-1
kDa
-72
LIMK-1
kDa
-72
p-CFL-1
kDa
-17
CFL-1
kDa
-17
GAPDH
kDa
-34

Fig S6B

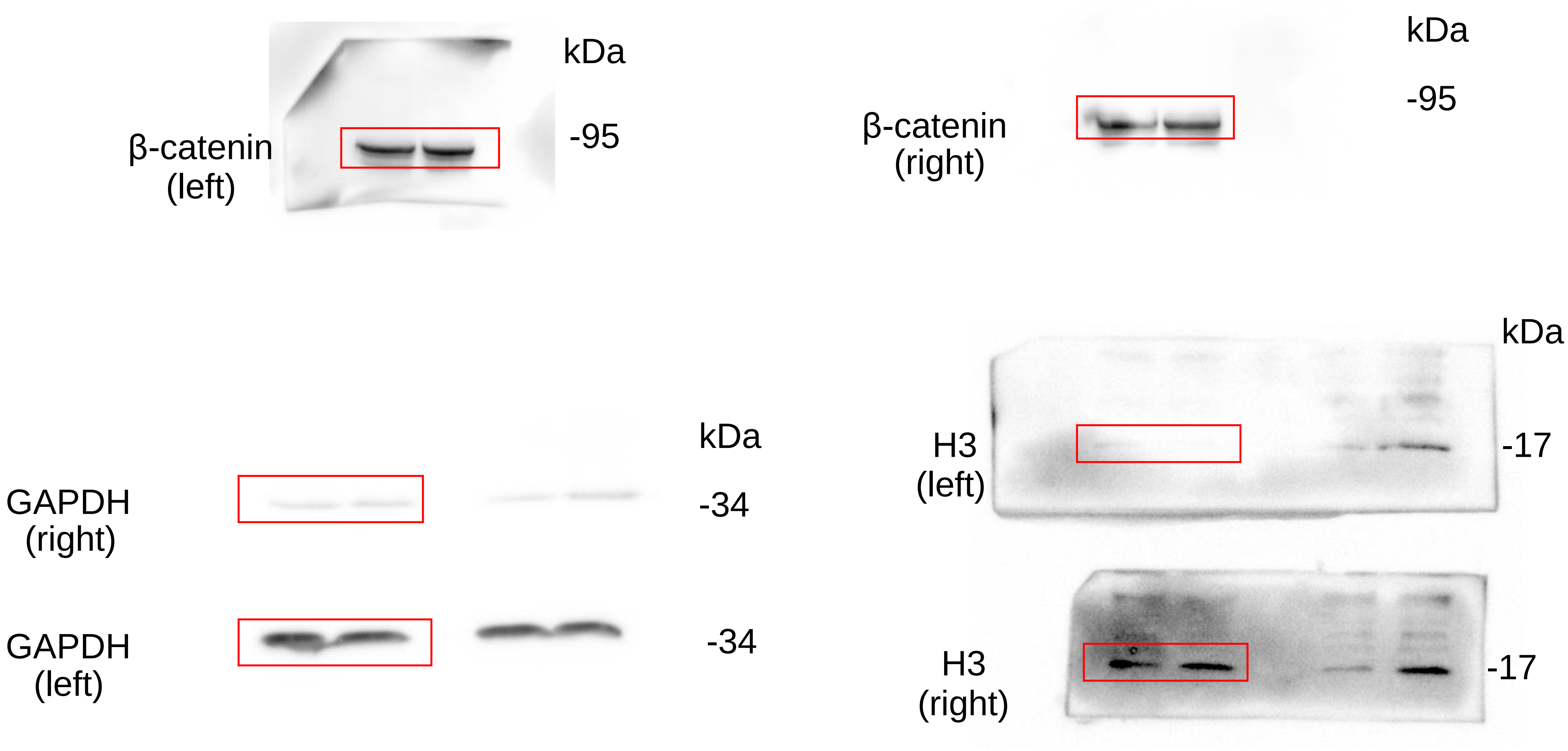

β-catenin
(left)
kDa
-95
β-catenin
(right)
kDa
-95
GAPDH
(right)
GAPDH
(left)
kDa
-34
-34
H3
(left)
H3
(right)
kDa
-17
-17
