# Supplementary material for: HUNK inhibits epithelial-mesenchymal transition of CRC via direct phosphorylation of GEF-H1 and activating RhoA/LIMK-1/CFL-1
Source: Cell Death Dis. 2023 May 16;14(5):327. doi: 10.1038/s41419-023-05849-2 (PMC10188538; doi:10.1038/s41419-023-05849-2)
Supplement: Supplementary file 4 — Supplementary Information -final [file 41419_2023_5849_MOESM4_ESM.docx]

**Supplementary** **Information**

**HUNK inhibits epithelial-mesenchymal transition of CRC via direct phosphorylation of GEF-H1 and activating RhoA/LIMK-1/CFL-1**

Xiaoqi Han^1,2,3^, Siyuan Jiang^4^, Yinmin Gu^4^, Lihua Ding^5^, Enhao Zhao^6^, Dongxing Cao^6^, Xiaodong Wang^7^, Ya Wen^1,2^, Yongbo Pan^2^, Xin Yan^2^, Liqiang Duan^2^, Minxuan Sun^7^, Tao Zhou^7^, Yajuan Liu^2^, Hongbo Hu^8^
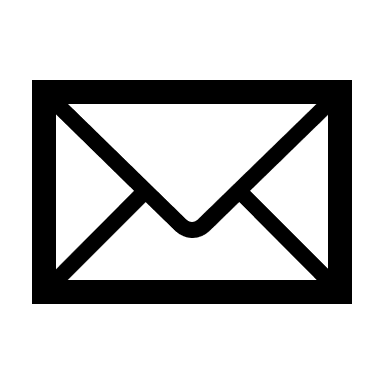
, Qinong Ye^5^
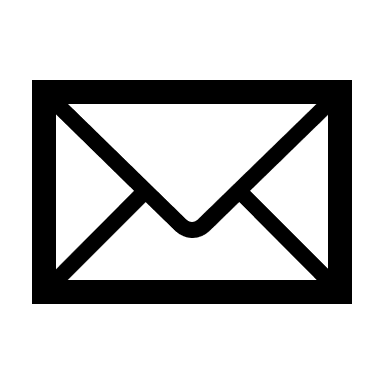
, Shan Gao^3^
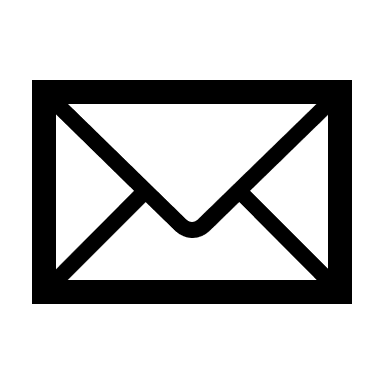


^1^Medical School of Guizhou University, Guiyang, 550025, China; ^2^Shanxi Academy of Advanced Research and Innovation, Taiyuan 030032, China; ^3^Zhongda Hospital, School of Life Sciences and Technology, Advanced Institute for Life and Health, Southeast University, Nanjing 210096, China; ^4^Zhongda Hospital, Medical School, Advanced Institute for Life and Health, Southeast University, Nanjing 210096, China; ^5^Department of Medical Molecular Biology, Beijing Institute of Biotechnology, Collaborative Innovation Center for Cancer Medicine, Beijing, 100850, China; ^6^Renji Hospital, School of Medicine, Shanghai Jiaotong University, 201200, China; ^7^Suzhou Institute of Biomedical Engineering and Technology, Chinese Academy of Sciences, Suzhou 215163, China, ^8^Center for Immunology and Hematology, State Key Laboratory of Biotherapy, National Clinical Research Center for Geriatrics, West China Hospital, Sichuan University, and Collaborative Innovation Center for Biotherapy, Sichuan, 610044, China.


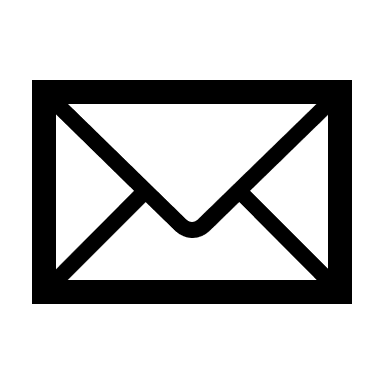
Corresponding author: Shan Gao, Zhongda Hospital, School of Life Sciences and Technology, Advanced Institute for Life and Health, Southeast University, Nanjing 210096, China. E-mail: gaos@sibet.ac.cn; Qinong Ye, Department of Medical Molecular Biology, Beijing Institute of Biotechnology, Collaborative Innovation Center for Cancer Medicine, Beijing, 100850, China. E-mail: yeqn88@163.com; Hongbo Hu, Center for Immunology and Hematology, State Key Laboratory of Biotherapy, National Clinical Research Center for Geriatrics, West China Hospital, Sichuan University, and Collaborative Innovation Center for Biotherapy, Sichuan, 610044, China. E-mail: hongbohu@scu.edu.cn.

This supplementary information contains:

- 23 Pages
- Supplementary Materials and Methods
- Supplementary Figures (6 Figures)
- Supplementary Tables (4 Tables)
- References

**Supplementary** **Materials** **and Methods**

**Data collection**

HUNK expression data and clinical data were downloaded from TCGA CRC project. The public database Gene Expression Omnibus (GEO) (1), Accession No. GSE131418 for CRC tissues were used.

**RNA-sequence and analysis**

Total RNAs were extracted from cells using Trizol Reagent (Thermo Fisher, AM8740) and sent to Novogene Technology Co., Ltd. (Beijing, P.R. China) for high-throughput sequencing. Raw read counts were used for differential gene expression analysis by DESeq2 (2, 3). The RNA sequencing data from this study have been deposited in the GEO (GSE 203440).

**Cell Culture**

Human CRC cells (SW480 and RKO) and HEK293T cells were grown in complete Dulbecco’s modified Eagle’s medium (DMEM; Hyclone) supplemented with 10% fetal bovine serum (FBS, Hyclone) and 1% penicillin/streptomycin (Hyclone). Human CRC cell lines were obtained with informed consents from ATCC (American Type Culture Collection). All cell lines were authenticated with short tandem repeats (STR) profiling and tested for mycoplasma contamination every month using MycoAlert (Lonza).

**siRNAs**

The sequences of siRNA for KD used are listed in Table S3.

**Immunoblot**

Cells were washed in cold phosphate buffer solution (PBS), homogenized and lysed in a RIPA buffer containing 50 mM Tris-HCl pH 8.0, 150 mM NaCl, 1% NP-40, 0.5% Nadeoxycholate, 0.1% Sodium dodecyl sulfate (SDS). After quantification using a BCA protein assay kit (Beyotime Biotechnology). Total protein was subjected to SDS-PolyAcrylamide Gel Electrophoresis (PAGE) and transferred to PolyVinylideneFluoride (PVDF) membrane (Millipore). The membrane was blocked for 1 h in wash buffer (PBS containing 0.1% Tween 20) containing 5% non-fat dry milk or Gelatin from porcine skin (Beyotime Biotechnology) followed by an overnight incubation with primary antibody diluted at 4°C. After extensive washing, the membrane was incubated with secondary antibody for 1 h in blocking buffer, washed, and processed using the immobilon western horseradish peroxidase substrate kit (Millipore). The antibodies used were given in Table S4.

**Transwell migration and invasion assays**

Transwell assays was conducted according to previously described protocol (4). 6.5 mm Transwell® with 8.0 µm Pore Polycarbonate Membrane Inserts (Corning) were used for migration assays, and 6.5 mm Corning® BioCoat™ Matrigel® Invasion Chambers (Corning) were used for invasion assays. 2×10^5^ cells in serum-free medium were seeded into the upper chamber, and the lower compartment was filled with normal culture medium supplemented with 20% FBS as the chemoattractant. Cells were incubated for 36 h for the migration assay and 48 h for the invasion assay. At the end of the experiments, the cells on the upper surface of the membrane were removed, and the cells on the lower surface were fixed and stained with 0.2% crystal violet. Three visual fields of each insert were randomly chosen and counted under a light microscope. Each condition was assayed in triplicate.

**Immunofluorescence**

Immunofluorescence was performed based on a universal protocol. Cells on glass coverslips (NEST) were fixed with cold methanol and permeabilized with 0.5% Triton X-100 in PBS. Samples were blocked in 1% donkey serum in the presence of PBS and stained with the appropriate primary and secondary antibodies coupled to Alexa Fluor 488 or 647 (Invitrogen). Confocal images were captured on a confocal microscope (Leica, Germany) with a ×63 oil objective.

**Immunoprecipitation**

Cell lysates were prepared by incubating the cells in IP buffer (50 mM tris-HCl (pH 7.5), 150 mM NaCl, 0.5% Nonidet P-40) in the presence of protease inhibitor 100× protease inhibitor Cocktail (Thermo Fisher Scientific) for 20 min at 4°C. This was followed by centrifugation at 14,000g for 15 min at 4°C. For immunoprecipitation, about 1 mg of protein and concentration 4 mg/ml was incubated with control or specific antibodies (2 μg) for 12 h at 4°C with constant rotation; 30 μl of protein A/G magnetic beads (Millipore) was then added and the incubation was continued for an additional 2 h. Beads were then washed five times using the lysis buffer. The precipitated proteins were boiled in 5 × loading buffer loading buffer and subjected to SDS-PAGE followed by immunoblotting with appropriate antibodies.

**Co-Immunoprecipitation**

Each group used 5 plates of 15 cm petri dish cells, about 5×10^8^ cells. Discard the cell culture medium and rinse the cells with pre-cooled PBS solution for three times. The cells were scraped off with a clean cell scraper and transferred to a 15 ml centrifuge tube. Centrifuge was used at 4℃ for 1000 rpm for 5 min to collect the cells. Add 5 ml IP cracking liquid suspension cells, and put it on 4 ℃ rotation meter cracking 30 min. The lysed cell solution was centrifuged at 15,000 rpm in a 4℃ centrifuge for 20 min to collect the supernatant, and 80 µl was removed from each group as Input; 100 µl anti- FLAG M2 magnetic beads (Thermo Fisher Scientific) cleaned with IP lysate were added to each group, and cell lysate and beads were placed on a rotator at 4℃ for 3-4 h; After the combination, centrifuge at 4℃ for 500 g for 5 min and discard the supernatant of cell lysate; 500 µl IP lysate was added, and the magnetic beads were transferred to a pre-cooled centrifuge tube. The magnetic beads were washed for 5 min on a rotator at 4℃, then centrifuged 500 g for 5 min in a 4℃ centrifuge, finally the supernatant was discarded. Repeat the previous step for eight times, and drain the washing liquid in the magnetic beads. 100 µl FALG peptide (Thermo Fisher Scientific, 500 µg/µl) diluted into 1× PBS was added to each group and placed in a rotator at 4℃ for rotating elution for 30 min, followed by centrifuge at 4℃ for 500 g for 5 min to collect the supernatant as elution sample. The elution samples were proportioned into 5 × loading buffer and boiled for 10 min at 100℃ for Western blotting analysis or frozen at -20℃ for storage.

**LC/MS Proteomics Data Analysis**

Gel pieces were cut from SDS PAGE, destained with 30% ACN/100 mM NH_4_HCO_3_ until the gels were destained. The gels were dried in a vacuum centrifuge. The in-gel proteins were reduced with dithiothreitol (10 mM DTT/ 100 mM NH_4_HCO_3_) for 30 min at 56° C, then alkylated with iodoacetamide (200 mM IAA/100 mM NH_4_HCO_3_) in the dark at room temperature for 30 min. Gel pieces were briefly rinsed with 100 mM NH_4_HCO_3_ and ACN, respectively. Gel pieces were digested overnight in 12.5 ng/μl trypsin in 25 mM NH_4_HCO_3_. The peptides were extracted three times with 60% ACN/0.1% TFA. The extracts were pooled and dried completely by a vacuum centrifuge. Each fraction was injected for nanoLC-MS/MS analysis. The peptide mixture was loaded onto a reverse phase trap column（Thermo Scientific Acclaim PepMap100, 100 μm × 2 cm, nanoViper C18）connected to the C18-reversed phase analytical column (Thermo Scientific Easy Column, 10 cm long, 75 μm inner diameter, 3μm resin) in buffer with 0.1% Formic acid and separated with a linear gradient of buffer with 84% acetonitrile and 0.1% Formic acid at a flow rate of 300 nl/min controlled by IntelliFlow technology. LC-MS/MS analysis was performed on a Q Exactive mass spectrometer (Thermo Scientific) that was coupled to Easy nLC (Proxeon Biosystems, now Thermo Fisher Scientific). The MS data were analyzed using MaxQuant software version 1.5.3.17 (Max Planck Institute of Biochemistry in Martinsried, Germany).

**Site-directed mutant molecular cloning**

The primers are designed according to the primer design principle. Generally, the front end of the coding sequence mutation site of the target gene is 15-20 bp and the back end is 15-20 bp as the front primer, and complementary reverse pairing is used as the back primer, and the corresponding enzyme digestion is added before and after the primers respectively. Sites and protected bases can be appropriately adjusted according to G/C content and annealing temperature. For the target DNA fragment that needs to be amplified, TransStart® FastPfu DNA high-fidelity polymerase is used for PCR amplification. Finally, the template plasmid is digested with FastDigest DpnI (Thermo Fisher).

***In vivo* metastasis analysis**

Animal experiments 5-week-old BALB/C nude mice were obtained from Vital River Laboratory (Beijing, China). Monitoring Institute approved all animal protocols used in this study. Mice were bred in specific pathogen free (SPF) animal house with 28 °C and 50% humidity. Cells (2 × 10^5^) were injected into the tail vein of nude mice to generate a metastasis model. After 30 days, the mice were dissected and the lung tissue was placed in tissue fixative; verified by hematoxylin and eosin (H&E) staining. For the STS experiment, 12 mice were injected with 2 × 10^5^ wild-type SW480 cells, and two days later, the mice were randomly divided into two groups of six mice, one group was injected with STS at a concentration of 3 mg/kg, and the other group was injected with an equal amount of placebo doses, treated for five consecutive days, and the mice were dissected after 30 days. All protocols involving animals were previously approved by the Ethics Committee for the Use of Experimental Animals of the Suzhou Institute of Biomedical Engineering and Technology, Chinese Academy of Sciences (Suzhou, Jiangsu, China).

**Protein purification**

E. coli BL21 strain was used to transform GST and target DNA plasmid, and cultured overnight. Monoclonal colonies were selected and inoculated in 500 ml bacterial medium, and cultured in 37℃ shaking table at 180 rpm for 12-16 h until the OD 600 nm value of bacterial solution was 0.6~0.8. Each tube was induced by adding 100 mM IPTG at 1:1000, and shaking at 180 rpm at 18℃ overnight. Centrifuge at 4,000 rpm for 10 min, discard the supernatant, configure bacterial lysis solution, add 1% NP-40 PBS solution of 100 × protease inhibitor Cocktail, and resuspend the bacterial solution under centrifugation. High pressure homogenizer was used to break the thallus. Supernatant was collected by centrifuge at 15,000 rpm at 4℃ for 1 min. Use 0.25 µm filter membrane to filter the centrifugal supernatant once to prevent the subsequent blocking of the binding column. 200 µl Glutathione Sepharose 4B agarose beads were added into each tube for rotation and binding at 4℃ for 1 h. 1 ml precooled bacterial lysate was washed with agarose beads for 5 times, centrifuged 500 g at 4℃ for 5 min, and the supernatant was completely absorbed. 20 µl of washed agarose beads were added into 5 × loading buffer for immunoblot electrophoresis analysis. Finally, the protein glue after electrophoresis was carefully removed and stained with coomassie bright blue solution at room temperature for more than 1 h to observe the protein purification results. The rest were added into the protease inhibitor cocktail and stored in a refrigerator at 4℃.

**GST pulldown**

After verifying the purified expression of the protein and the effect of *in vitro* translation of the protein, remove the frozen GST or recombinant proteins which have been combined with beads, add 4% BSA 20 µl, 100 × protease inhibitor Cocktail 4 µl, and supplement with PBS to the final volume of 100 µl. The swirl mixer was mixed and incubated at 4℃ for 30 min. The in vitro translation proteins were added to GST and the purified proteins, respectively, and were mixed and incubated at 4℃ for 2 h. Add 1 ml wash buffer beads: rotate on the whirlpool mixer at 4℃ for 5 min, centrifuge at 500 g at 4℃ for 5 min, repeat twice, wash with wash buffer three times; After the supernatant was discarded, protein loading buffer was added along with Input, and the sample was cooked at 95℃ for 10 min for immunoblot electrophoresis analysis and detection, or frozen at -20℃ for later use.

**TnT® Quick Coupled Transcription/Translation Systems**

Following the instructions, TnT® Quick Master Mix, Methionine (1Mm), DNA template and Nuclease-Free Water were mixed to then appropriate volume. The reaction was incubated at 30°C for 60–90 minutes. Then the proteins were purified for experiments.

***In Vitro* Protein Kinase Assay**

Human FLAG-tagged HUNK was purified from HEK293T cells using anti- FLAG M2 magnetic beads. GST-GEF-H1(623-684) and GEF-H1 S645A mutant were expressed in the E. coli BL21 strain. 2 μg of the indicated GST fusion proteins were incubated with the purified HUNK-FLAG in the kinase buffer (Cell Signaling Technologies) together with 200 μM cold ATP (Beyotime Biotechnology) for 1 h at 30°C. Reactions were stopped by addition of SDS loading buffer, and samples were then heated for 5 min at 95°C before analysis by SDS-PAGE.

**Anti-****p-GEF-H1(S645) antibody preparation**

Peptide synthesis and anti-p-GEF-H1(S645) antibody preparation were performed by GeneScript Biotechnology Co., Ltd (Nanjing). Briefly, a phosphopeptides LFRSE{pSer}LESPRGERC were synthesized, and polyclonal antibodies against the p-GEF-H1(S645) peptide were obtained from inoculated rabbits. Antibodies were purified using affinity chromatography on columns containing the corresponding peptides.

**LC/MS phosphorylomics**

For digestion, the co-IP eluent was reduced with 5 mM dithiothreitol for 30 min at 56 °C and alkylated with 11 mM iodoacetamide for 15 min at room temperature in darkness. The protein sample was then diluted by adding 100 mM TEAB to urea concentration less than 2M. Finally, trypsin was added at 1:50 trypsin-to-protein mass ratio for the first digestion overnight and 1:100 trypsin-to-protein mass ratio for a second 4 h digestion. After trypsin digestion, peptide was desalted by Strata X C18 SPE column (Phenomenex) and vacuum-dried. Peptide was reconstituted in 0.5 M TEAB and processed according to the manufacturer’s protocol for TMT kit/iTRAQ kit. The peptide mixtures were then incubated for 2 h at room temperature and pooled, desalted and dried by vacuum centrifugation. The tryptic peptides were fractionated into fractions by high pH reverse-phase HPLC using Betasil C18 column (5 μm particles, 10 mm ID, 250 mm length, Thermo). Briefly, peptides were first separated with a gradient of 8% to 32% acetonitrile (pH 9.0) over 60 min into 60 fractions. Then, the peptides were combined into 6 fractions and dried by vacuum centrifuging. Peptide mixtures were first incubated with IMAC microspheres suspension with vibration in loading buffer (50% acetonitrile/6% trifluoroacetic acid). The IMAC microspheres with enriched phosphopeptides were collected by centrifugation, and the supernatant was removed. To remove nonspecifically adsorbed peptides, the IMAC microspheres were washed with 50% acetonitrile/6% trifluoroacetic acid and 30% acetonitrile/0.1% trifluoroacetic acid, sequentially. To elute the enriched phosphopeptides from the IMAC microspheres, elution buffer containing 10% NH_4_OH was added and the enriched phosphopeptides were eluted with vibration. The supernatant containing phosphopeptides was collected and lyophilized for LC-MS/MS analysis. The tryptic peptides were dissolved in 0.1% formic acid (solvent A), directly loaded onto a home-made reversed-phase analytical column (15 cm length, 75 μm, Thermo). The peptides were subjected to NSI source followed by tandem mass spectrometry (MS/MS) in Q ExactiveTM Plus (Thermo) coupled online to the UPLC. The resulting MS/MS data were processed using Maxquant search engine (v.1.5.2.8). Tandem mass spectra were searched against human UniProt database concatenated with reverse decoy database.

**Human tissue and immunohistochemistry**

All procedures for collecting CRC specimens were approved by Renji Hospital, School of Medicine，Shanghai Jiaotong University (Shanghai, P.R. China) logic characteristics were obtained from medical records. All 58 patients provided informed consent. The study was approved by the Institutional Review Board (IRB) of Renji Hospital, School of Medicine, Shanghai Jiaotong University. Immunohistochemistry was performed based on a universal protocol (5). Briefly, paraffin-embedded slides were rehydrated, and the endogenous peroxidase was blocked by 3% H_2_O_2_ for 15 min at room temperature. Then, sections were incubated in retrieval buffer and boiled for 3.5 min. After 3 washes with PBS, slides were incubated with indicated antibodies (1:100/300 dilution) at 4°C overnight followed by treatment with MaxVision HRP solution for 30 min at room temperature. After 3 washes with PBS, sections were stained with 3,3N-Diaminobenzidine Tertrahydrochloride Peroxidase Substrate. Slides were imaged using fluorescence microscope. H-score is assigned using the following formula: [1 × (% cells 1+) + 2 × (% cells 2+) + 3 × (% cells 3+)] ×100.

**Cell proliferation assay**

The cells were seeded in 96-well plates, with each well containing 1500-2000 cells in 100 μl of cell suspension. After a certain time in culture, cell viability was measured using CellTiter-Glo Luminescent™ (CTG) Cell Viability Assay assay (Promega).

**Nuclear/cytosolic fractionation**

Cell nucleus/cytoplasm fraction isolation was performed according to previously described protocol (6). 1×10^7^ HUNK KO and wild type cells were incubated with hypotonic buffer (25 mM Tris-HCl, PH 7.4, 1 mM MgCl2, 5 mM KCl) on ice for 5 minutes. An equal volume of hypotonic buffer containing 1% NP-40 was then added, and each sample was left on ice for another 5 minutes. After centrifugation at 5000 g for 5 minutes, the supernatant was collected as the cytosolic fraction. The pellets were resuspended in nucleus resuspension buffer (20 mM HEPES, PH 7.9, 400 mM NaCl2, 1 mM EDTA, 1 mM EGTA, 1 mM DTT, 1 mM PMSF), and incubated at 4°C for 30 minutes. Nuclear fraction was collected after removing insoluble membrane debris by centrifugation at 12000 g for 10 minutes.

**Quantification and statistical analysis**

Data were presented as the mean ± standard error of the mean (SEM) or standard deviation (SD). Two-tailed Student's t-test for individual comparison. Dunnett test and Tukey test for multiple comparisons were performed to assess the statistical significance of differences between groups. Spearman's correlation was performed to analyse the correlation. *p* value of < 0.05 was considered statistically significant. All statistical analyses were performed using GraphPad Prism 8.0 or R software (version 3.5.2) (5).

**Supplementary Figures**


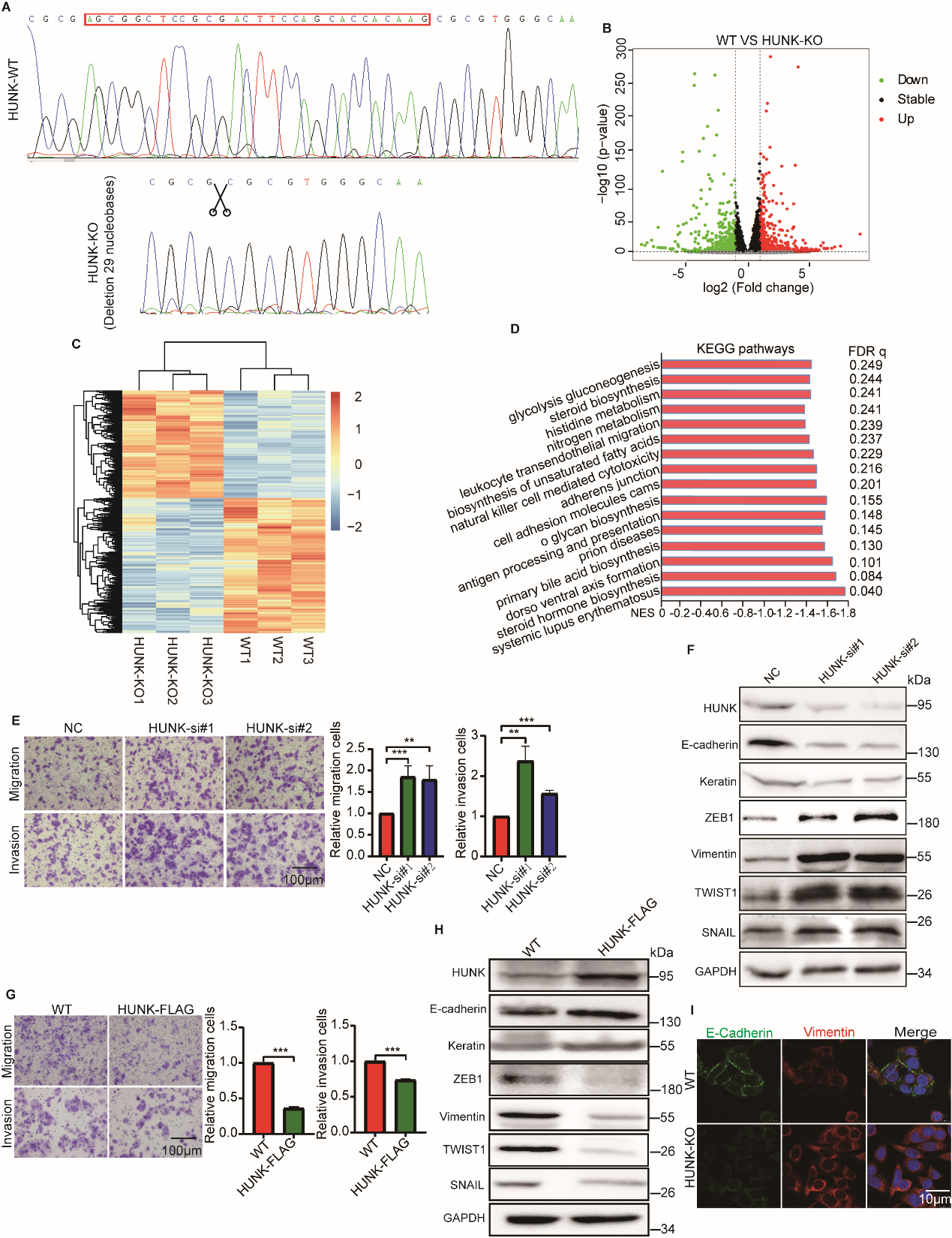


**Figure S1. HUNK suppresses EMT of CRC cells.** (A) DNA sequence analysis of HUNK KO clone in SW480 cells. (B) Volcano plot showing the downregulated 535 (green) and upregulated 289 (red) genes in RNA-seq data of HUNK KO SW480 cells. (C) Heatmap showing differentially expressed genes (DEGs) upon HUNK KO SW480 cells. Color represents the levels of DEGs. (D) The top 16 enriched KEGG terms for DEGs. (E and G) Representative micrographs (left) and quantification (right) of the HUNK KD (E) and OE (G) RKO cells in Matrigel-coated or noncoated Transwell assays (n=3). (F and H) Immunoblot analysis of the indicated proteins in HUNK KD (F) and OE (H) RKO cells. (I) Immunofluorescence of E-cadherin and Vimentin in HUNK KO and WT SW480 cells. Nuclei are labeled with DAPI. The scale bar represents 10 μm. Results are presented as the mean ± SEM. One-way ANOVA with Dunnett's multiple comparisons test was applied for analyzing the data in (E), Two-tailed Student's t-test was used for analyzing the data in (G). ***p* <0.01, and ****p* < 0.001.


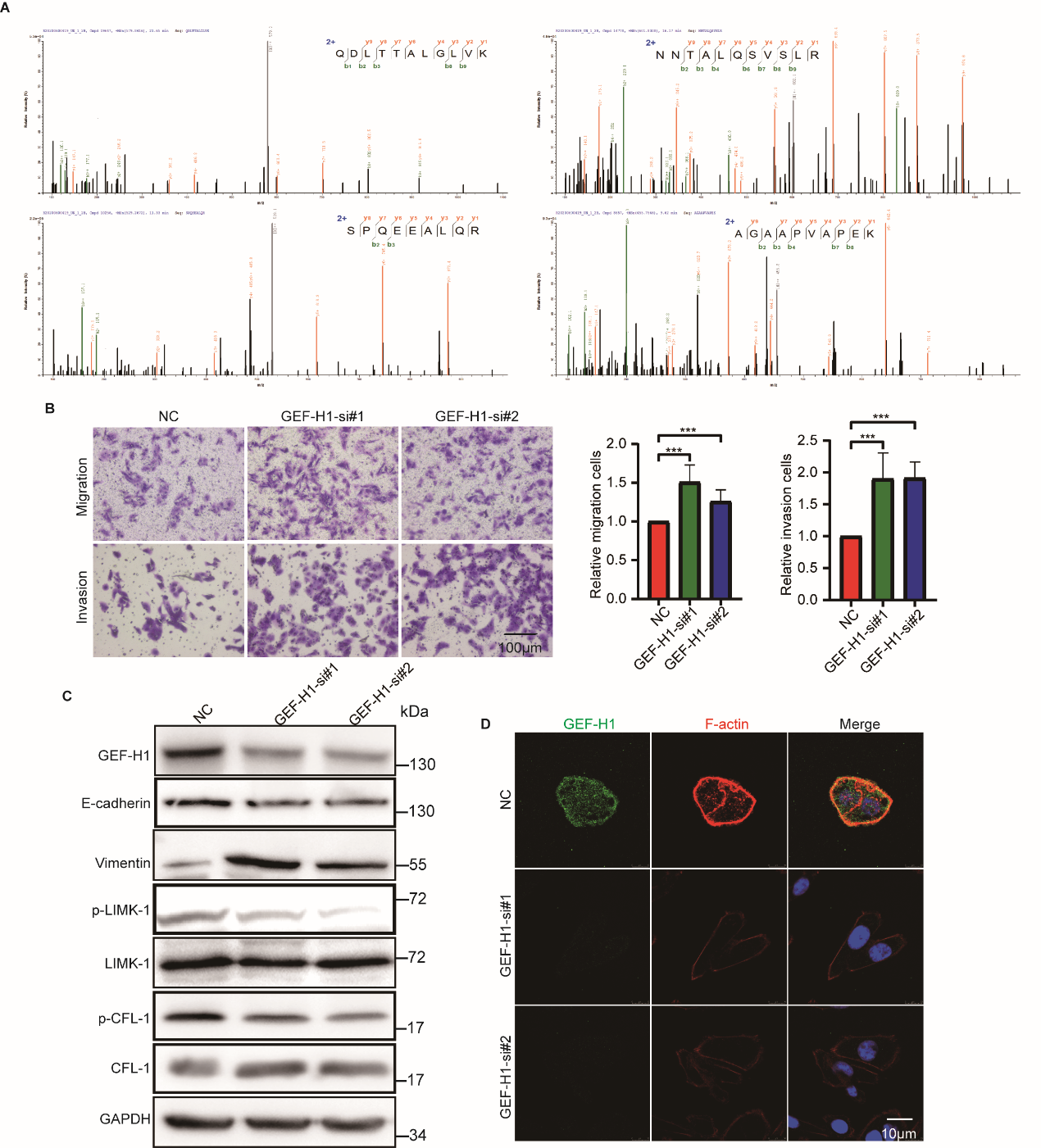


**Figure S2. GEF-H1 as a** **suppressor of EMT of CRC cells.** (A) LC-MS analysis of the HUNK-FALG associated peptides corresponding to GEF-H1. (B) Representative micrographs (left) and quantification (right) of the GEF-H1 KD SW480 cells in Matrigel-coated or noncoated Transwell assays (n=3). Scale bars, 100 μm. (C) Immunoblot analysis of the indicated proteins in SW480 GEF-H1 KD SW408 cells. (D) F-actin and GEF-H1 were stained in GEF-H1 KD in SW480 cells. Nuclei labeled with DAPI. The scale bar represents 10 μm. Results are presented as the mean ± SEM. One-way ANOVA with Dunnett's multiple comparisons test was applied for analyzing the data in (B). ****p* < 0.001.


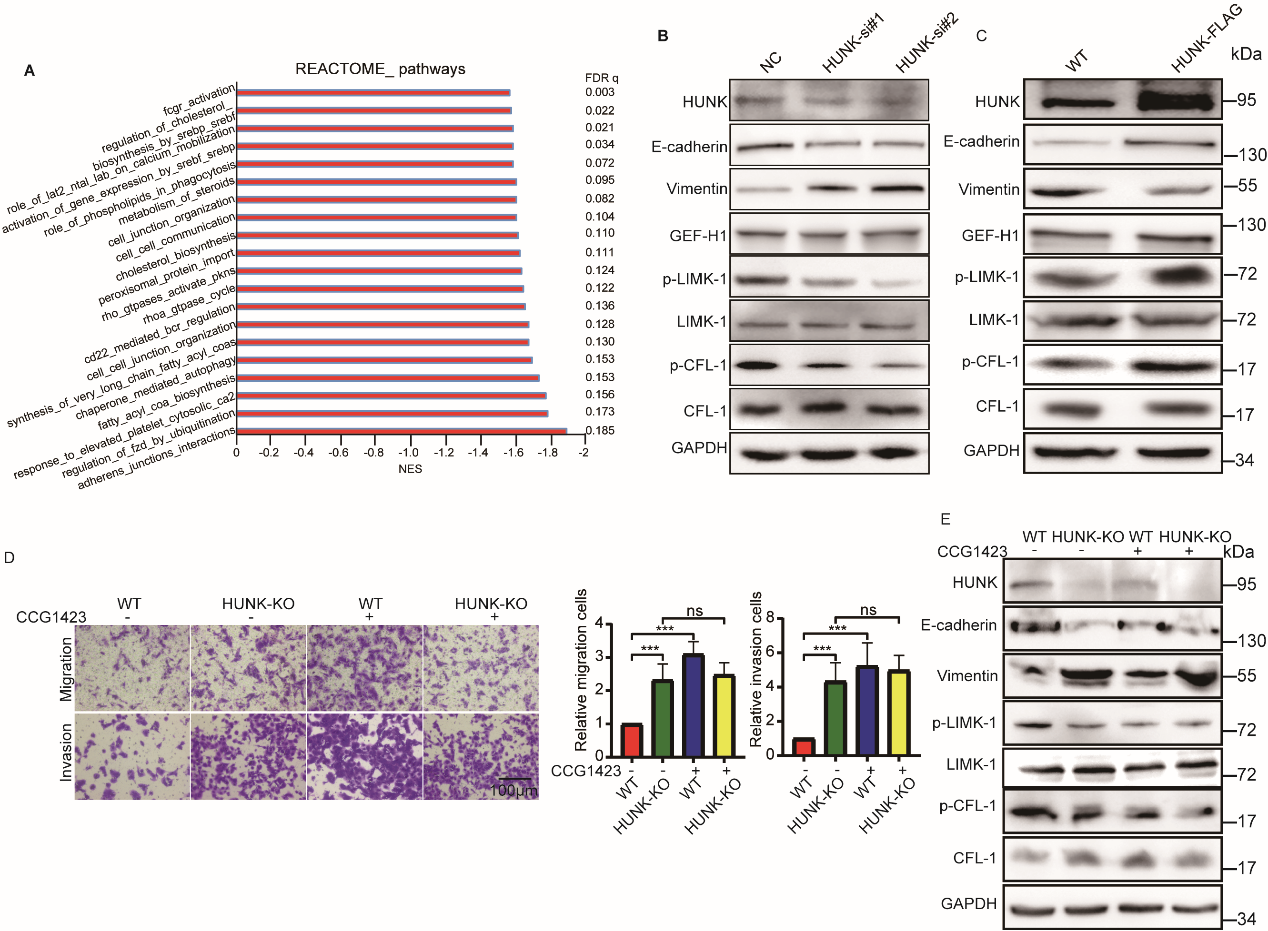


**Figure S3. HUNK inhibits RhoA/LIMK-1/CFL-1 signaling pathway activity.** (A) The top 20 enriched REACTOME terms for HUNK KO SW480 cells. (B-C) Immunoblot analysis of the indicated proteins in RKO HUNK KD (B), and OE (C) cells. (D) Representative micrographs (left) and quantification (right) of the CCG-1423 treated SW480 cells in Matrigel-coated or noncoated Transwell assays (n=3), Scale bars, 100 μm. (E) Immunoblot analysis of the indicated proteins in CCG-1423 treated-SW480 cells. Results are presented as the mean ± SEM. One-way ANOVA with Tukey's multiple comparisons test was applied for analyzing the data in (D). ****p* < 0.001. ns, not significant.


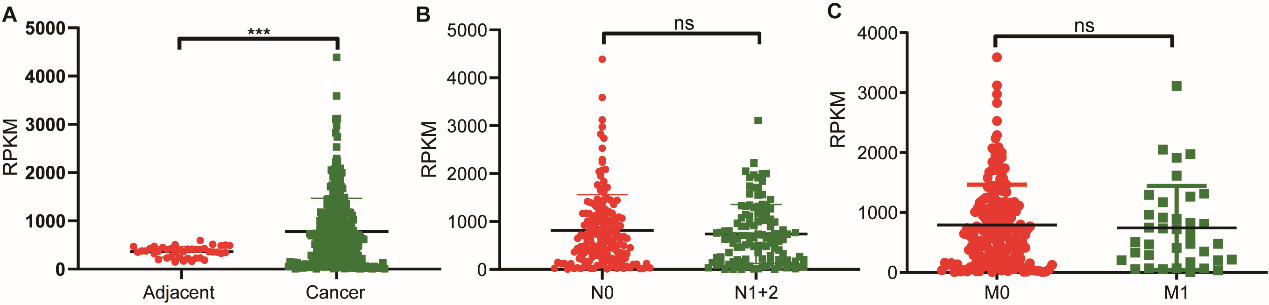
 **Figure S4. The clinical relevance of the HUNK mRNA levels**. (A) The expression levels of HUNK in CRC tissues (green) and adjacent (red) tissues based on TCGA database. (B-C) The expression levels of HUNK in node (N0 versus N1+2) and metastatic (M0 versus M1) tissues of CRC patients. Results are presented as the mean ± SEM. Two-tailed Student's t-test was used for analyzing the data in (A-C). ****p* < 0.001. ns, not significant.


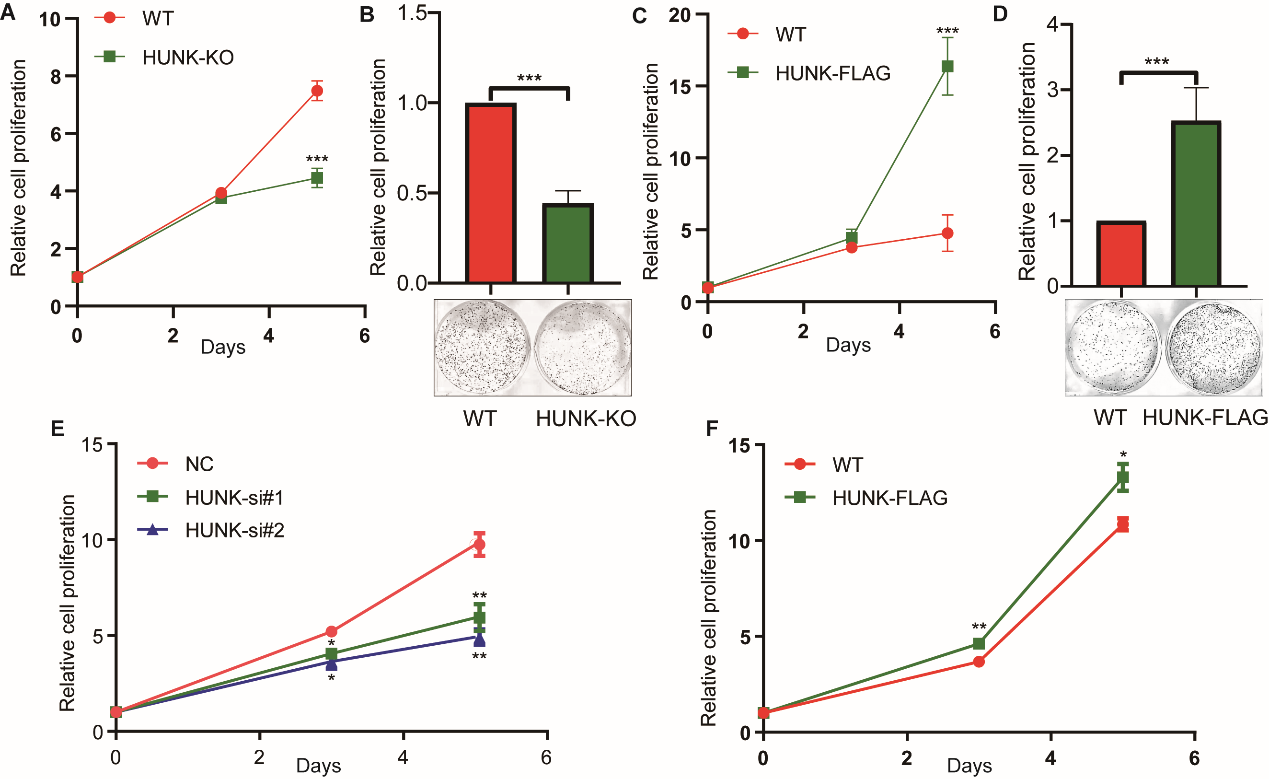
 **Figure S5. HUNK promotes CRC cell proliferation**. (A) The CTG assay for the cell proliferation in HUNK-KO of SW480 cells (n=3). (B) Representative images of colony formation assay (bottom) and quantification data (top) for HUNK-KO of SW480 cells (n=3). (C) The CTG assay for the cell proliferation in HUNK OE of SW480 cells (n=3). (D) Representative images of colony formation assay (down) and quantification data (top) in HUNK OE of SW480 cells (n=3). (E-F) The CTG assay for the cell proliferation in HUNK KD and OE of RKO cells (n=3). Results are presented as the mean ± SEM. Two-tailed Student’s t-test was used for analyzing the data in (A-D) and (F). One-way ANOVA with Dunnett's multiple comparisons test was applied for analyzing the data in (E). ***p* < 0.01 and ****p* < 0.001.


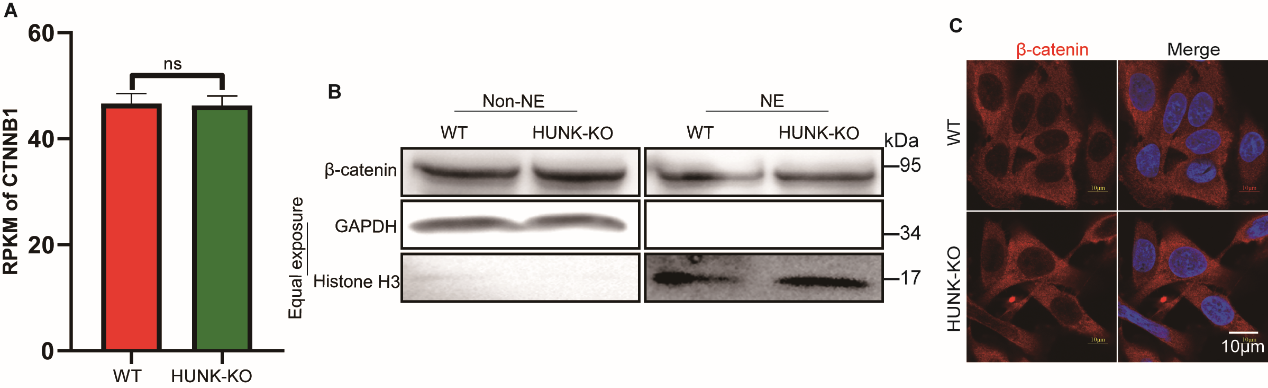


**Figure S6. HUNK effects on the expression level and localization of β-catenin**. (A) Expression level of *CTNNB1* (encoding β-catenin) in WT (red) and HUNK KO (green) SW480 cells based on HUNK KO cell RNA expression profile. (B) Immunonlot analysis of β-catenin expression for the indicated proteins in subcellular fractions. GAPDH and Histone H3 served as the cytoplasmic and nuclear markers for the whole-cell lysates (WCL), cytoplasmic (Non-NE) and nuclear (NE) fraction, respectively. (D) β-catenin was stained in WT and HUNK KO SW480 cells. Nuclei labeled with DAPI. The scale bar represents 10 μm. Results are presented as the mean ± SD. Two-tailed Student’s t-test was used for analyzing the data in (A). ns, not significant.

**Supplementary Tables**

**Table S1 Mass spectrometry analysis of HUNK-associated proteins.**

| **Accession** | **Protein Identity** | **# Unique Peptides** | **# Peptides** | **# PSMs** |
| --- | --- | --- | --- | --- |
| P57058 | HUNK | 28 | 28 | 199 |
| P63151 | PP2A subunit B | 6 | 14 | 1 |
| Q92974 | GEF-H1 | 4 | 4 | 4 |

**Table S2 Clinicopathologic characteristics of CRC patients**

| **Characteristics** | **Number of cases** |
| --- | --- |
| **M classification** | |
| M0 18 | |
| M1 | 40 |

**Table S3 Sequences of siRNA for KD.**

| **Targets** | **RNA-Forward (5'-3')** | **RNA-Reverse (5'-3')** |
| --- | --- | --- |
| si-NC | UUCUCCGAACGUGUCACGUTT | ACGUGACACGUUCGGAGAATT |
| si-HUNK #1 | GCUACUAGAUGAAGACAAUTT | AUUGUCUUCAUCUAGUAGCTT |
| si-HUNK #2 | GCCAUCUACUUCCUCUUAATT | UUAAGAGGAAGUAGAUGGCTT |
| si-GEF-H1 #1 | CCCUGUACUUGAGUUUCAATT | UUGAAACUCAAGUACAGGGTT |
| si-GEF-H1 #2 | CCAAGUACCCGUUACUCAUTT | AUGAGUAACGGGUACUUGGTT |

**Table S4 List of primary antibodies.**

| Antibodies | Manufacturer | Application |
| --- | --- | --- |
| HUNK | Abacam, #ab189059 | 1:300 for IHC |
| HUNK | Abacam, #ab137492 | 1:1000 for WB |
| GAPDH | Proteintech, #60004-1-Ig | 1:5000 for WB |
| E-cadherin | Cell Signaling Technology, # 14472 | 1:1000 for WB |
| Keratin | Beyotime Biotechnology, # AG2429 | 1:1000 for WB |
| ZEB1 | Cell Signaling Technology, # 83243 | 1:1000 for WB |
| Vimentin | Abacam, #ab92547 | 1:2000 for WB |
| SNAIL | Cell Signaling Technology, #3879 | 1:1000 for WB |
| TWIST1 | Beyotime Biotechnology, # AF8274 | 1:1000 for WB |
| GEF-H1 | Abacam, #50599-2-Ig | 1:1000 for WB, 3μg for IP |
| PP2A B Subunit | Cell Signaling Technology, # 4953S | 1:1000 for WB, 3μg for IP |
| FLAG | Cell Signaling Technology, # 14793 | 1:3000 for WB |
| HA | Cell Signaling Technology, # C29F4 | 1:3000 for WB |
| RhoA | Abacam, # ab187027 | 1:1000 for WB |
| p-LIMK-1 | Cell Signaling Technology, # 3841S | 1:1000 for WB |
| LIMK-1 | Cell Signaling Technology, # 3842S | 1:1000 for WB |
| p-CFL-1 | Cell Signaling Technology, # 3313S | 1:1000 for IF |
| CFL-1 | Cell Signaling Technology, # 5175S | 1:1000 for WB |
| HIS | Cell Signaling Technology, # 12698S | 1:1000 for WB |
| p-GEF-H1(S645) | Genscript, #Custom antibody | 1:1000 for WB,1:200 for IHC |
| Phosphoserine | Abacam, # ab232944 | 1:500 for WB |
| β-catenin | Cell Signaling Technology, # 8480 | 1:1000 for WB |
| Histone H3 | Beyotime Biotechnology, # AF0009 | 1:1000 for WB |
| Phalloidin | Mesgenbio, # MF8203 | 1mg/ml for IF |

**References**

1. Clough E, Barrett T. The Gene Expression Omnibus Database. Methods In Molecular Biology (Clifton, NJ). 2016;1418.

2. Love MI, Huber W, Anders S. Moderated estimation of fold change and dispersion for RNA-seq data with DESeq2. Genome Biol. 2014;15(12):550.

3. Yang X, Wen Y, Liu S, Duan L, Liu T, Tong Z, et al. LCDR regulates the integrity of lysosomal membrane by hnRNP K-stabilized LAPTM5 transcript and promotes cell survival. Proc Natl Acad Sci U S A. 2022;119(5).

4. Gu Y, Niu S, Wang Y, Duan L, Pan Y, Tong Z, et al. DMDRMR-Mediated Regulation of m(6)A-Modified CDK4 by m(6)A Reader IGF2BP3 Drives ccRCC Progression. Cancer Res. 2021;81(4):923-34.

5. Wang XD, Yang XH, Zhang C, Wang Y, Cheng TY, Duan LQ, et al. Tumor cell-intrinsic PD-1 receptor is a tumor suppressor and mediates resistance to PD-1 blockade therapy. P Natl Acad Sci USA. 2020;117(12):6640-50.

6. Wang J, Yang X, Li R, Wang L, Gu Y, Zhao Y, et al. Long non-coding RNA MYU promotes prostate cancer proliferation by mediating the miR-184/c-Myc axis. Oncology Reports. 2018.
